# ARPC2: A Pan-Cancer Prognostic and Immunological Biomarker That Promotes Hepatocellular Carcinoma Cell Proliferation and Invasion

**DOI:** 10.3389/fcell.2022.896080

**Published:** 2022-06-06

**Authors:** Shenglan Huang, Cairong Dong, Dan Li, Yongkang Xu, Jianbing Wu

**Affiliations:** ^1^ Department of Oncology, The Second Affiliated Hospital of Nanchang University, Nanchang, China; ^2^ Jiangxi Key Laboratory of Clinical and Translational Cancer Research, Nanchang, China; ^3^ Department of Hepatobiliary Surgery, The Second Affiliated Hospital of Nanchang University, Nanchang, China

**Keywords:** ARPC2, prognostic biomarker, immune infiltrating, pan-cancer, hepatocellular carcinoma

## Abstract

**Background:** Actin-related protein 2/3 complex subunit 2 (ARPC2) plays a fundamental role in actin filament nucleation and is critical for tumor cell migration and invasion. However, its abnormal expression, clinical significance, and biological function in human pan-cancer have been poorly explored. Thus, we focused on ARPC2 as an entry point for identifying novel pan-cancer prognostic biomarkers.

**Methods:** The Cancer Genome Atlas (TCGA) and the Genotype-Tissue Expression (GTEx) databases were used to assess the differential expression of ARPC2 in pan-cancer. The Human Protein Atlas was used for the tissue/cell-specific expression analysis of ARPC2. The genetic alteration information of ARPC2 was obtained from the cBioPortal database and the GSCALite platform. The prognostic value of ARPC2 was explored in pan-cancer using Cox regression and Kaplan–Meier analyses. Spearman correlation analysis was performed to investigate the relationship between ARPC2 expression and tumor mutational burden (TMB), DNA methyltransferases, microsatellite instability (MSI), immune-related genes, and mismatch repairs (MMRs). The ESTIMATE and CIBERSORT algorithms were used to evaluate the association between ARPC2 expression and the tumor microenvironment (TME) and immune infiltrating cells. We also conducted differential expression analysis of ARPC2 in hepatocellular carcinoma (HCC) tissues and cell lines using qPCR, western blotting, and immunohistochemistry and explored its role in tumor proliferation, migration, and invasion of HCC cells.

**Results:** ARPC2 expression was significantly upregulated in multiple tumor types and significantly correlated with worse prognosis and higher clinicopathological stage. Genetic alterations and DNA methylation in tumor tissues may contribute to the aberrant expression of ARPC2. ARPC2 expression was significantly correlated with the tumor microenvironment (TME), infiltrating immune cells, TMB, microsatellite instability (MSI), and immune checkpoint-related genes in certain cancer types. In this experimental study, we found that the expression of ARPC2 was dramatically upregulated in HCC tissues and cell lines compared to adjacent liver tissues and normal liver cell lines. Functionally, ARPC2 silencing in HCC cells significantly inhibited cell proliferation, migration, and invasion, while the overexpression of ARPC2 promotes tumor proliferation, migration, and invasion.

**Conclusion:** ARPC2 is a promising prognostic and immunological biomarker for multiple tumor types and is likely to play an important role in HCC progression and metastasis.

## Introduction

Cancer is a major public health problem and a leading cause of death worldwide (Siegel et al., 2021). The World Health Organization reported 19.3 million new cancer cases and almost 10.0 million deaths by cancer in 2020 (Sung et al., 2021). The expected global cancer burden will increase, with the projected prevalence reaching 28.4 million cases by 2040, representing a 47% increase from 2020 (Sung et al., 2021). Lung cancer ranks first in terms of cancer-related mortality (18%), followed by colorectal (9.4%), liver (8.3%), stomach (7.7%), and female breast (6.9%) cancers (Sung et al., 2021). Over the past 10 years, the application of immunotherapy has resulted in groundbreaking advances in cancer treatment. For example, immune checkpoint inhibitors (ICIs) can induce durable tumor remission in a wide variety of cancers by modulating the immune system (Hiam-Galvez et al., 2021). Nevertheless, most immunotherapies are largely beneficial for patients with specific genetic mutations and certain tumor types, and their efficacy remains limited in most clinical settings (Haslam and Prasad, 2019). Moreover, biomarker-matched trials related to immunotherapy are still limited in most cancers, and many genomic alterations remain challenging to target (Khemlina et al., 2017). Thus, there is an urgent need to identify more specific immune biomarkers and explore the association between cancer and immunology. More importantly, it is imperative to identify new gene alteration targets involved in tumorigenesis and tumor development to discover new therapies for malignancies.

Actin-related protein 2/3 complex subunit 2 (ARPC2) is a core component of the actin-related protein 2/3 (Arp2/3) complex and stabilizes the complex, which is involved in the regulation of actin polymerization. The Arp2/3 complex is essential for cell division, migration, endocytosis, membrane trafficking, phagocytosis, and pathogenic infection (Yoon et al., 2019). ARPC2 promotes cancer cell proliferation and invasion by regulating the expression of oncogenes or tumor suppressor genes in various cancers, including breast (Cheng et al., 2019), pancreatic (Rauhala et al., 2013), and gastric cancers (Zhang J et al., 2017). In our recent study, we used bioinformatics analysis and reported that ARPC2 expression is associated with poor prognosis in patients with HCC (Huang et al., 2021). Moreover, ARPC2 inhibitors (benproperine and pimozide) can significantly suppress the migration and invasion of cancer cells and hamper tumor metastasis in animal models (Choi et al., 2019; Yoon et al., 2019). Therefore, ARPC2 has been suggested as a potential biomarker for cancer diagnosis and therapy. However, the expression level and clinical significance of ARPC2 in most cancer types remain elusive, and its biological role remains to be elucidated.

In this study, we comprehensively explored the expression pattern of ARPC2 in 33 human cancer types and different cell lines based on The Cancer Genome Atlas (TCGA), the Genotype-Tissue Expression (GTEx) database, and the Human Protein Atlas (HPA) website. The association of ARPC2 with prognostic and clinicopathological parameters was analyzed in pan-cancer based on the UCSC Xena database. In addition, we investigated the genetic alteration features of ARPC2 in various cancers, including the tumor mutational burden (TMB), DNA methylation, microsatellite instability (MSI), and mismatch repairs (MMRs). Moreover, we focused on the analysis of the correlation between ARPC2 expression and the tumor microenvironment (TME) and tumor immunity in pan-cancer. In addition, we conducted a series of experiments to determine the expression levels of APRC2 in HCC cell lines and tissues and to explore its role in tumor proliferation, migration, and invasion in HCC cells.

## Materials and Methods

### Tissue Sample Collection and Ethics Approval

We collected 42 paired HCC tissues and adjacent noncancerous tissues following informed written consent from all patients involved in the study; the consent also pertained to the publication of the results. Patients with HCC who underwent tumor surgical resection at the Second Affiliated Hospital of Nanchang University (Nanchang, China) between November 2020 and November 2021 were included in the study. The tissues were stored in a refrigerator at −80°C until further analysis. The study was conducted in accordance with the Declaration of Helsinki of the World Medical Association and approved by the Second Affiliated Hospital of Nanchang University Medical Research Ethics Committee.

### Pan-Cancer Data Collection and Expression Analysis of ARPC2 Pan-Cancer

RNA sequencing data in the transcripts per million reads format (TPM) of 33 human cancer types from TCGA and GTEx datasets were downloaded from the UCSC Xena database (https://xenabrowser.net/datapages/), including 10,534 pan-cancer samples from TCGA datasets and 15,776 samples from TCGA-integrated GTEx datasets. The sequencing data were normalized to log2 (TPM +1). Clinical information, including survival time, survival status, histological types, and tumor-node-metastasis (TNM) stage, was also acquired from the UCSC Xena database (http://xena.ucsc.edu/).

We extracted the pan-cancer expression value of ARPC2 using the “limma” package in R 4.0.5 (http:///www.r-project.org/). We used the Wilcoxon rank sum test to compare the expression level of ARPC2 in tumor tissues and the corresponding normal tissues using “ggpubr” package in R 4.0.5, and a *p*-value <0.05 was considered to be statistically significant. We further explored the mRNA expression specificity of ARPC2 in different tissues, single-cell types, and blood cell lineages from HPA (https://www.proteinatlas.org/). The results were directly obtained from the HPA database by typing the gene name in the search box, and then, by sequentially selecting the modules of “tissue,” “single-cell types,” and “immune cell.”

### Genetic Mutation Analysis

GSCALite (Liu et al., 2018) is an online platform for gene set cancer analysis that integrates cancer genomic data of 33 cancer types from TCGA. In this study, we downloaded genetic alteration information of ARPC2 pan-cancer from the cBioPortal database (https://www.cbioportal.org/) *via* the “Cancer Types Summary” and “Mutations” modules; the data retrieved included the alteration frequency, mutation type, copy number alteration, and posttranslational modification (PTM) sites. We also utilized the GSCALite web server (Liu et al., 2018) (http://bioinfo.life.hust.edu.cn/web/GSCALite/) to explore the association of ARPC2 expression with gene copy number variations (CNVs) and DNA methylation state based on Pearson’s product-moment correlation coefficient. At least five genes were required to perform this analysis; therefore, we entered Arp2/3 complex genes and highlighted the results of ARPC2 with black boxes. Finally, according to gene co-expression in TCGA datasets, we conducted Spearman correlation analysis to assess the relationship of ARPC2 expression with five methyltransferases (DNMT1, DNMT3A, DNMT3B, DNMT3L, and TRDMT1) and five MMR-related genes (*MLH1*, *MSH2*, *MSH6*, *PMS2*, and *EPCAM*) in 33 types of cancer. Statistical significance was determined with a *p*-value <0.05.

### Prognostic Analysis and Correlation of ARPC2 Expression With Clinical Characteristics

To explore the prognostic value of ARPC2 pan-cancer, we first integrated the expression data and clinical information of TCGA datasets and removed data with incomplete survival time and survival status information. Then, the Kaplan–Meier method and univariate Cox regression analysis were used to explore the correlation of ARPC2 expression with overall survival (OS), disease-specific survival (DSS), and progression-free interval (PFI) in pan-cancer using “survival” and “survminer” packages of R 4.0.5. Patients with different types of cancer were assigned to high- and low-expression groups according to the best statistical cutoff values, and the log-rank test and Cox regression were used to calculate the significance of survival times between the two subgroups. The prognostic results were presented as hazard ratios (HRs), 95% confidence intervals, and *p-*values, and statistical significance was obtained when *p* < 0.05. Thereafter, the ARPC2 expression levels in different clinical characteristics, including tumor size (T1–2 *vs.* T3–4), TNM stage (stage I–II *vs.* stage III–IV), and different histological types, were analyzed using the “limma” and “ggpubr” R packages. We used the Wilcoxon rank sum test to compare the gene expression level in different clinicopathological characteristics, and *p*-values <0.05 were considered significant. The differences with *p* < 0.05 are displayed with box plots (*p* < 0.05).

### Correlation of ARPC2 With the TME and Tumor Immunity

First, we applied the ESTIMATE algorithm (Yoshihara et al., 2013) to calculate the stromal and immune scores in 10,328 tissues from 33 types of cancer using the “estimate” R package, which represents the abundance of stromal cells and infiltrating immune cells in the tumor microenvironment (TME). The ESTIMATE scores are the sum of the stromal and immune scores and can be used to infer tumor purity. Correlation scores were then calculated to determine the relationships between the stromal/immune/ESTIMATE scores and ARPC2 expression using the Spearman method. The analyses were implemented in “ggplot2,” “ggpubr,” and “ggExtra” packages in R. The top 10 cancer types with the most significant association between ARPC2 expression and the TME are presented in the Results section, and the correlations were visualized using scatterplots. Thereafter, we downloaded immune cell infiltration estimations for 33 cancer types from the TIMER2.0 database (http://timer.comp-genomics.org/), and CIBERSORT (https://cibersort.stanford.edu/) was used to calculate the relative proportions of 22 infiltrating immune cells in each patient. Spearman’s correlation analyses were performed to describe the relationship between ARPC2 expression and the proportion of these immune cells. A *p*-value <0.05 was considered statistically significant in the aforementioned analyses.

### Correlation Between ARPC2 Expression and Treatment Response to ICIs

Recently, ICIs have been widely regarded as one of the most promising approaches for cancer therapy, and their use has resulted in the unprecedented extension of patient survival for a wide array of cancer types (Bagchi et al., 2021). Previous studies have indicated that immune checkpoint-related genes are associated with the efficacy of ICIs (Nishino et al., 2017). TMB and MSI have been proposed as predictive biomarkers of response to ICIs (Schrock et al., 2019; McGrail et al., 2021). In this study, the associations between ARPC2 expression and 47 immune checkpoint-related genes were assessed using Spearman’s correlation coefficient and visualized with a heatmap. We also calculated TMB of each patient based on the exome sequencing data from TCGA using “varscan 2” and obtained summarized MSI data of 33 cancer types from previous studies (Hause et al., 2016; Yang et al., 2019). Correlation analyses between TMB/MSI and ARPC2 expression were performed using the Spearman’s test and visualized as radar plots. A *p*-value <0.05 was considered statistically significant.

### Cell Culture

Four human HCC cell lines (HCC-LM3, MHCC97-H, HepG2, and huh-7) were purchased from Procell Life Science and Technology Co., Ltd. (Wuhan, China), and one normal liver cell line, L-02, was obtained from the Cell Bank of the Chinese Academy of Science (Shanghai, China). All cells were cultured in high-glucose Dulbecco’s modified Eagle’s medium (DMEM) supplemented with 10% fetal bovine serum (FBS; Gibco, Grand Island, NY, United States), 100 μg/ml streptomycin, and 100 U/ml penicillin sodium (Biotechnology, Beijing, China). The cells were incubated in a humidified incubator with 5% CO_2_ at 37°C. Subsequently, the mRNA and protein expression levels of ARPC2 in each cell line were detected using real-time reverse transcription quantitative polymerase chain reaction (RT-qPCR) and western blot assays, respectively. The L-02 cell line served as a control.

### Transfection

We designed three different small interfering RNA (siRNA) sequences to silence ARPC2 in HCC cell lines. The siRNAs-targeting ARPC2 (si-ARPC2#1, si-ARPC2#2, and si-ARPC2#3) and control siRNA (si-NC) were synthesized by Hanheng Biological Technology (Shanghai, China). The pcDNA 3.1(+) vector was used to construct the ARPC2 overexpression plasmid (OE-ARPC2) and the control overexpression plasmid (OE-NC). The plasmids were purchased from Hanheng Biological Technology (Shanghai, China). The siRNA transfection was performed using TransIntroTM EL Transfection Reagent (TransGen Biotech, Beijing, China), according to the manufacturer’s instructions. The OE-ARPC2 and OE-NC plasmids were transfected into HCC cell lines using Lipofectamine 3000 transfection reagent (Invitrogen; Thermo Fisher Scientific, Inc.), according to the manufacturer’s instructions. After 48 h of transfection, the knockdown and overexpression efficiencies were measured using RT-qPCR and western blotting. The transfected HCC cells were collected for subsequent functional experiments. The siRNA sequences are listed in the supplementary material (Supplementary Table S1).

### RT-qPCR

Total RNA was dissociated from HCC cells and tissue samples using TRIzol reagent (Invitrogen, Carlsbad, CA, United States), according to the manufacturer’s protocol. Then, the RNA was reverse-transcribed to complementary DNA (cDNA) using the PrimeScript™ RT reagent kit with gDNA Eraser (RR047A, TaKaRa, China). qPCR was performed with TB Green^®^ Premix Ex Taq™ II (RR820A, TaKaRa, China) on a CFX96 real-time PCR detection system. The reaction conditions were 94.0°C for 30 s, followed by 94.0°C for 4 s, 58.0°C for 15 s, and 72°C for 15 s, for a total of 40 cycles. The relative ARPC2 mRNA expression in HCC cells was calculated using the 2^−ΔΔCt^ method, whereas the expression in HCC tissue samples was calculated using 2^−ΔCt^. The expression levels were normalized to those of the endogenous control glyceraldehyde 3-phosphate dehydrogenase (GAPDH). The primers used are listed in Supplementary Table S1.

### Western Blot Analysis

We first extracted total protein from HCC cells and tissues using radioimmunoprecipitation assay (RIPA) lysis buffer (BB-3209, Bestbio Science, Beijing, China), according to the manufacturer’s instructions. The BCA assay kit (Beyotime, Shanghai, China) was used to determine the protein concentration. Subsequently, equal amounts of protein were separated using 10% sodium dodecyl sulfate-polyacrylamide gel electrophoresis (SDS-PAGE) at a constant voltage (200 V), and then transferred to a PVDF membrane at 260 mA electricity for 2 h. Subsequently, the membrane was blocked with 5% nonfat milk for 2 h at 37°C and incubated with rabbit antihuman ARPC2 antibody (1:2,000, ab133315, Abcam). GAPDH (1:5,000, 60004-1-Ig, Proteintech) was used as an internal reference. After being incubated overnight with the primary antibody at 4°C, the membrane was incubated with horseradish peroxide (HRP)-conjugated secondary antibodies (1:10,000, SA00001-1, SA00001-2, Proteintech) at room temperature for 1.5 h. After washing the protein bands with 1× TBST buffer three times for 30 min, color rendering was performed using ECL chemiluminescence (Beyotime Biotechnology, China). Image Lab analysis software (version 4.0, Bio-Rad) and ImageJ (ImageJ 1.8.0) were used to quantitatively assess the relative protein abundance by gray-scale scanning.

### Immunohistochemistry Analysis

The fresh tissue samples of both tumor and adjacent noncancerous tissues were collected immediately after resection and fixed in 10% formalin. Then, they were embedded in paraffin, cut into 3–4-μm thick pieces, planked on a glass slide, and baked at 60°C for 2 h. The slides were then dewaxed with xylene and hydrated with an alcohol gradient. The endogenous peroxidase activity was blocked with 0.3% H_2_O_2_ for 20 min. After antigen retrieval, the slides were incubated overnight with primary rabbit antihuman ARPC2 antibodies (1:500, ab133315, Abcam) at 4°C, followed by incubation at 37°C with secondary antihorseradish peroxidase antibodies for 30 min. Tissue sections were stained with hematoxylin and visualized with 3,3′-diaminobenzidine (DAB). Finally, after dehydration and sealing, the images were obtained using light microscopy. We further calculated the average optical density (mean density) of each image using Image-Pro Plus 6.0 (Media Cybernetics, Inc., Rockville, MD, United States) and compared the ARPC2 expression between HCC tissues and adjacent noncancerous tissues using an unpaired *t*-test.

### Cell Proliferation Assays and Flow Cytometry Analysis

Cell proliferation was detected using 5-ethynyl-2′-deoxyuridine (EdU) staining assays. First, the HCC cells (2 × 10^5^/ml), which were transfected with siRNA fragments or overexpression plasmids for 48 h, were collected and uniformly seeded in 96-well plates with 100 μl culture medium containing 10% FBS. After being cultured for another 24 h, the cells were subjected to the EdU assay using an EdU kit (C6016L, US Everbright^®^ Inc., China), according to the manufacturer’s instructions. In brief, the HCC cells were labeled using the EdU staining kit for 2 h at 37°C and fixed with 4% paraformaldehyde for 20 min at room temperature. Next, the cells were incubated with 2 mg/ml glycine for 5 min, followed by washing with 3% bovine serum albumin. The cells were then permeabilized with 0.1% Triton X-100, and EdU staining reagents were used according to the manufacturer’s instructions. Positive EdU-stained cells were observed under a fluorescence microscope, and the percentage of proliferating cells was calculated using ImageJ software (ImageJ 1.8.0).

Cell apoptosis was measured using the FITC-annexin V and PI apoptosis kits (F6012, US Everbright^®^ Inc., China). According to the kit manual, the transfected cells seeded in 6-well plates (approximately 2.5 × 10^5^ cells/well) were digested with EDTA-free trypsin (Solarbio Biotechnology, Beijing, China) and washed three times with phosphate-buffered saline (PBS). Then, 100 μl of mixed buffer was added to resuspend the cells, and the cell density was adjusted to 1 × 10^5^ ml. Next, 5 μl of FITC-annexin V and 5 μl PI were added to the cell suspension. After incubation in the dark for 15 min at 4°C, 400 μl of mixed buffer was added to the mix reaction. The percentage of apoptotic cells, including early and late apoptotic ones, was examined using a FACSCalibur flow cytometer (BD Biosciences).

### Scratch and Transwell Assays

Scratch assays were performed to determine cell migration. First, the transfected HCC cells were uniformly seeded into 6-well plates and cultured until they reached 100% confluency. Then, the cell monolayer was lightly scratched using a sterile 200-μl pipette tip and washed with PBS (Solarbio Biotechnology, Beijing, China) to remove floating cells. Then, fresh medium was added, and culturing was continued for 48 h. At 0, 24, and 48 h after cell scratching, cell migration was observed at the same site under a microscope, and the scratch area was measured using ImageJ. HCC cell migration was calculated using the following formula: cell migration rate (%) = (1 − scratch area/original scratch area) × 100%.

Transwell assays were performed to examine cell migration and invasion. Transwell chambers pre-coated with Matrigel (YB356234, BD Biosciences, United States) were used for the invasion assay, and chambers without Matrigel were used for the migration assay. Prior to cell collection, the Matrigel gel was diluted with a serum-free culture medium at a ratio of 1:8. Next, 60 μl of diluted Matrigel was added to the upper chamber, which was placed in a 24-well plate at 37°C for 2–3 h until curding. Next, the transfected cells (2 × 10^4^ cells) in 200 μl serum-free DMEM were added to the upper chamber, and 600 μl DMEM containing 10% FBS was added to the lower chamber. After being cultured for 48 h at 37°C, the HCC cells in the chamber were fixed with 4% formaldehyde for 15 min at room temperature and stained with 0.1% crystal violet at room temperature for 15 min. The cells on the upper surface of the chamber (unmigrated cells) were erased using a wet cotton swab. The cell migration or invasion was measured and photographed under an inverted microscope.

### Statistical Analysis

Bioinformatic analyses were performed using R software (https://www. r-project. org/, version 4.0.4). The Wilcoxon rank sum test was used to compare gene expression differences between the two groups, and the Kruskal–Wallis test was used for multiple group comparisons. The Kaplan–Meier method and Cox regression analysis were employed for survival assays. Spearman or Pearson’s correlation analyses were performed to clarify the correlation between the groups. GraphPad Prism 8.0 software was used for experimental statistical analysis. Unpaired *t*-tests were used for the comparisons between the two groups, and one-way analysis of variance was used for the comparison among multiple groups. All quantitative experiments were repeated three times, and the results are reported as mean ± standard deviation (SD). All statistical tests were two-sided, and statistical significance was set at *p* < 0.05.

## Results

### Expression Levels of ARPC2 in Human Pan-Cancer, Normal Tissues, and Cell Lines

**FIGURE 1:**
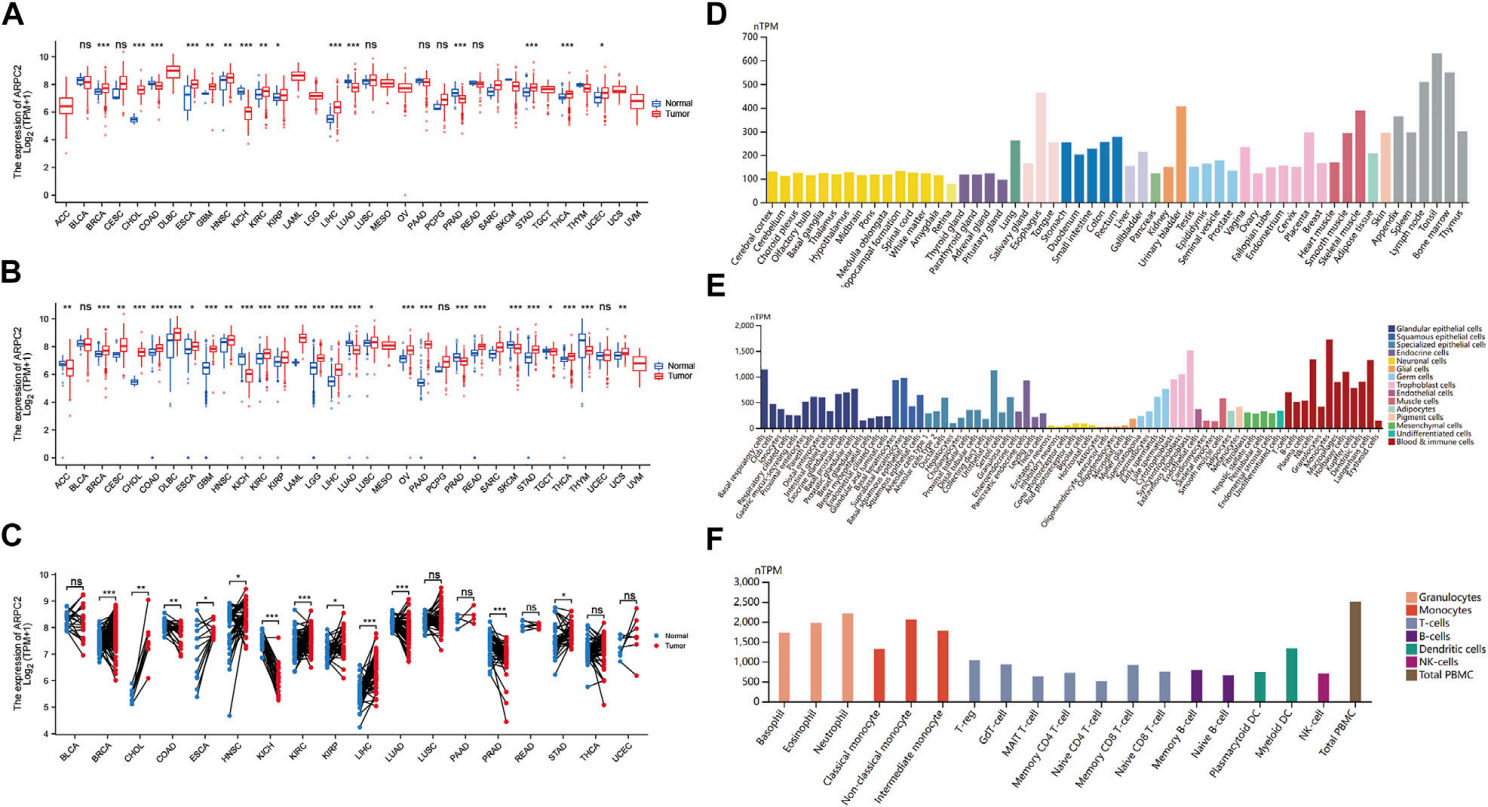
Differential expression of APRC2. **(A)** ARPC2 expression in pan-cancer tissues from TCGA database. **(B)** ARPC2 expression in pan-cancer tissues based on TCGA and GTEx databases. **(C)** ARPC2 expression in paired tumor and adjacent normal tissues from TCGA database. **(D)** ARPC2 mRNA expression in different tissues from the HPA database. **(E)** ARPC2 mRNA expression in different single-cell types from the HPA database. **(F)** ARPC2 mRNA expression in blood cell lineage from the HPA database. ns, no significance; *p < 0.05; **p < 0.01; and ***p < 0.001.

Transcriptome data of 33 cancer types were downloaded from UCSC Xena. The number of patients with the 33 primary tumors is displayed in Table 1. We extracted the mRNA expression of ARPC2 and analyzed the differential gene expression between the tumor and normal tissues using box plots. As shown in Figure 1A, based on the analysis of 11,903 samples from TCGA, ARPC2 mRNA expression was significantly upregulated in 11 cancer types: BRCA, CHOL, ESCA, GBM, HNSC, KIRC, KIRP, LIHC, STAD, THCA, and UCEC, compared with that in normal tissues. A significant downregulation of ARPC2 expression was observed in COAD, KICH, LUAD, and PRAD, and no statistically significant changes were observed in the case of BLCA, CESC, LUSC, PAAD, PCPG, and READ. Considering the absence of corresponding normal tissues in some cancer types, we integrated the GTEx and TCGA databases, including 15,775 samples, to examine the expression patterns of ARPC2 in the 33 cancer types. ARPC2 was also differentially expressed in ACC, DLBC, LAML, LGG, OV, TGCT, and UCS (Figure 1B). Furthermore, paired sample analysis was conducted on 18 cancer types, and the results indicated that the mRNA expression of ARPC2 increased significantly in BRCA, CHOL, ESCA, HNSC, KIRC, KIRP, LIHC, and STAD tissues, whereas it was downregulated in COAD, KICH, LUAD, and PRAD (Figure 1C).

**TABLE 1:** Tumor types and number of patients from TCGA and GTEx databases.

Abbreviation	Tumor type	Number of patients (TCGA)		Number of patients (TCGA + GTEx)	
		Normal	Tumor	Normal	Tumor
ACC	Adrenocortical carcinoma	0	77	128	77
BLCA	Bladder urothelial carcinoma	19	407	28	407
BRCA	Breast invasive carcinoma	113	1,099	292	1,099
CESC	Cervical squamous cell carcinoma and endocervical adenocarcinoma	3	306	13	306
CHOL	Cholangiocarcinoma	9	36	9	36
COAD	Colon adenocarcinoma	41	209	349	209
DLBC	Lymphoid neoplasm diffused large B cell lymphoma	0	47	444	47
ESCA	Esophageal carcinoma	13	182	666	182
GBM	Glioblastoma multiforme	5	166	1,157	166
HNSC	Head and neck squamous cell carcinoma	44	520	44	520
KICH	Kidney chromophobe	25	66	53	66
KIRC	Kidney renal clear cell carcinoma	72	531	100	531
KIRP	Kidney renal papillary cell carcinoma	32	289	60	289
LAML	Acute myeloid leukemia	0	173	70	173
LGG	Brain lower grade glioma	0	523	1,152	523
LIHC	Liver hepatocellular carcinoma	50	371	160	371
LUAD	Lung adenocarcinoma	59	515	347	515
LUSC	Lung squamous cell carcinoma	50	498	338	498
MESO	Mesothelioma	0	87	0	87
OV	Ovarian serous cystadenocarcinoma	0	427	88	427
PAAD	Pancreatic adenocarcinoma	4	179	171	179
PCPG	Pheochromocytoma and paraganglioma	3	182	3	182
PRAD	Prostate adenocarcinoma	52	499	152	499
READ	Rectum adenocarcinoma	10	167	318	167
SARC	Sarcoma	2	263	2	263
SKCM	Skin cutaneous melanoma	1	471	813	471
STAD	Stomach adenocarcinoma	32	375	210	375
TGCT	Testicular germ cell tumors		156	165	156
THCA	Thyroid carcinoma	58	510	338	510
THYM	Thymoma	2	119	446	119
UCEC	Uterine corpus endometrial carcinoma	35	552	101	552
UCS	Uterine carcinosarcoma	0	56	78	56
UVM	Uveal melanoma	0	80	0	80

The HPA website was used to investigate the mRNA expression levels of APRC2 in different human tissues and cell lines. The results demonstrated that ARPC2 is widely expressed in different human tissues, single-cell types, and blood cell lineages. The expression specificity was low in different tissues and cell lines. ARPC2 expression was relatively higher in the bone marrow, lymph nodes, and blood and immune cells, but lower in brain tissues and neuronal cells (Figures 1D–F).

### ARPC2 Genetic Alteration and DNA Modification in Pan-Cancer

**FIGURE 2:**
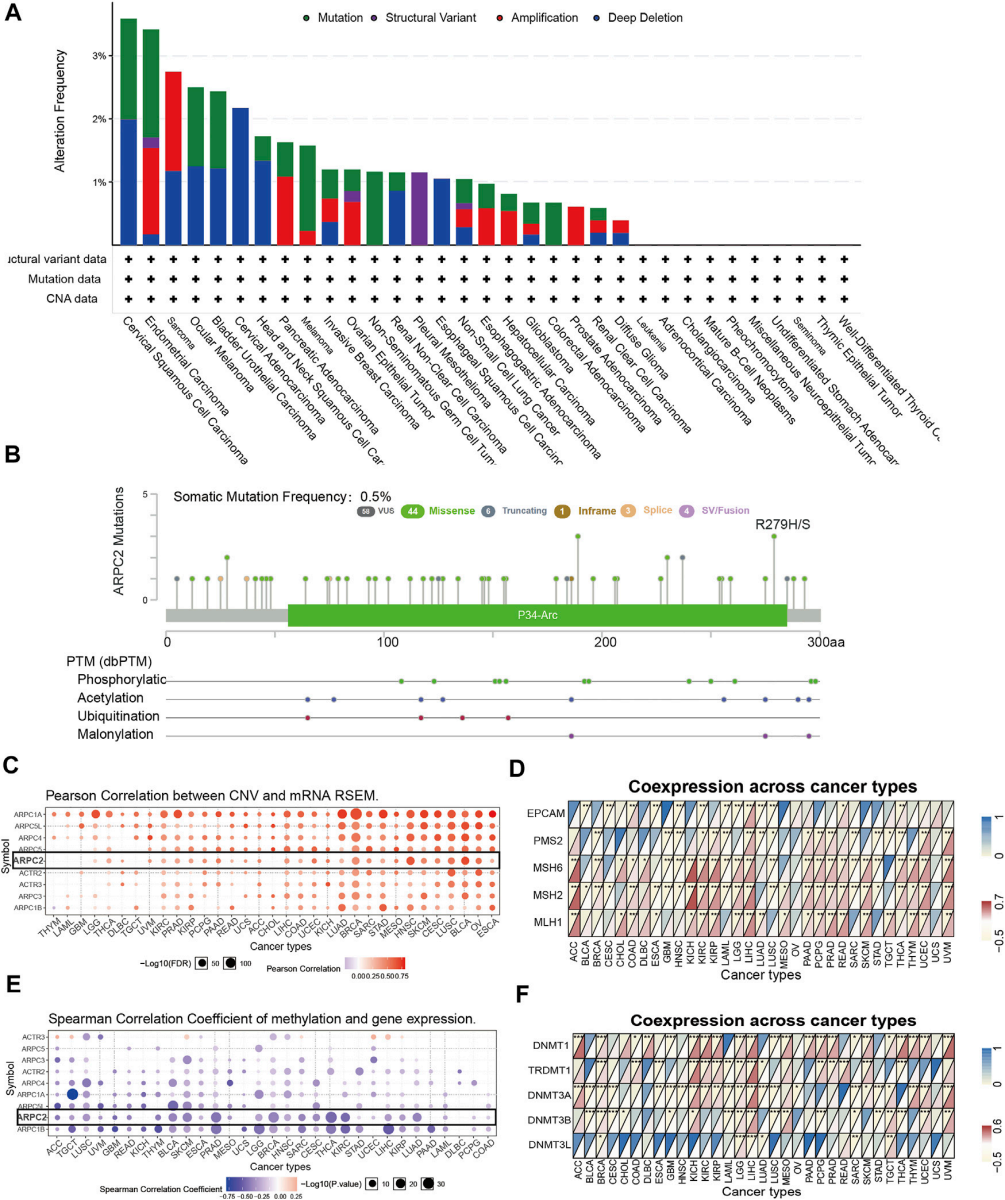
Genetic alteration and DNA modification of ARPC2 in pan-cancer. **(A)** Mutation type and frequency of ARPC2 obtained from the cBioPortal website. **(B)** Somatic mutation frequency and posttranslational modification sites of ARPC2. **(C)** Correlation between Arp2/3 complex expression and CNV in pan-cancer obtained from the GSCALite platform, black box represents ARPC2. **(D)** Correlation between ARPC2 expression and five MMRs related genes in 33 types of cancer. **(E)** Correlation between Arp2/3 complex expression and DNA methylation in pan-cancer obtained from the GSCALite platform, black box represents ARPC2. **(F)** Correlation between the expression of ARPC2 and five methyltransferases in 33 types of cancer. CNV, copy number variations; MMRs, mismatch repair. *p < 0.05; **p < 0.01; and ***p < 0.001.

To investigate the genetic alteration status of ARPC2 in pan-cancer, the online analysis platforms cBioPortal and GSCALite were used to analyze the gene alteration frequency, mutation type, and PTM sites. The results showed that the highest gene alteration frequency of ARPC2 occurred in cervical squamous cell carcinoma (3.59%), in which four cases (1.59%) had “mutation” and five cases (1.99%) had “deep deletion.” “Mutation” occurred most frequently in endometrial carcinoma (1.71%), while “structural variant,” “amplification,” and “deep deletion” often occurred in pleural mesothelioma (1.15%), sarcoma (1.57%), and cervical adenocarcinoma (2.17%) (Figure 2A). The frequency of somatic mutations was 0.5%, of which 44 cases (75.8%) showed missense mutations. The phosphorylation, acetylation, ubiquitination, and malonylation sites of ARPC2 are shown in Figure 2B. Pearson correlation analysis based on GSCALite showed that the ARPC2 expression levels were positively correlated with the CNV percentage in 28 cancer types (Figure 2C).

It is well known that MMR-related genes with aberrant expression are among the leading causes of somatic mutations, some of which eventually lead to tumorigenesis (Baretti and Le, 2018). Therefore, we evaluated the relationship between ARPC2 and MMR-related genes (MLH1, MSH2, MSH6, PMS2, and EPCAM). As shown in Figure 2D, ARPC2 expression had a significantly positive correlation with five MMR-related genes in HCC, and significant correlations were found between ARPC2 and at least one MMR-related gene in 29 other cancer types, except for OV and UCS. Positive associations were observed in 14 cancer types (ACC, COAD, KICH, KIRC, KIRP, LGG, LIHC, PAAD, PCPG, READ, SKCM, THYM, UCEC, and UVM) and a negative relationship was observed in ESCA, GBM, HNSC, LAML, LUSC, SARC, and STAD.

DNA methylation can regulate gene expression by affecting chromatin structure, DNA conformation, and stability as well as the interaction between DNA and DNA-binding proteins. Therefore, we explored the association between ARPC2 and DNA methylation. Pearson’s correlation analysis demonstrated that the promoter methylation level of ARPC2 was negatively linked to ARPC2 expression in 27 tumor types (Figure 2E), indicating that low DNA methylation might be one of the causes of high ARPC2 expression pan-cancer. In addition, we evaluated the relationship between ARPC2 expression and methyltransferases (DNMT1, TRDMT1, DNMT3A, DNMT3B, and DNMT3L). As shown in Figure 2F, ARPC2 expression was more closely related to DNMT1, which showed a positive correlation in 19 types of cancer and an inverse correlation in four cancer types, but was less closely associated with DNMT3L. ARPC2 expression was positively associated with all five methyltransferases in LGG but did not correlate with any of the five methyltransferases in USC.

### Prognostic Value of ARPC2 in Pan-Cancer

**FIGURE 3:**
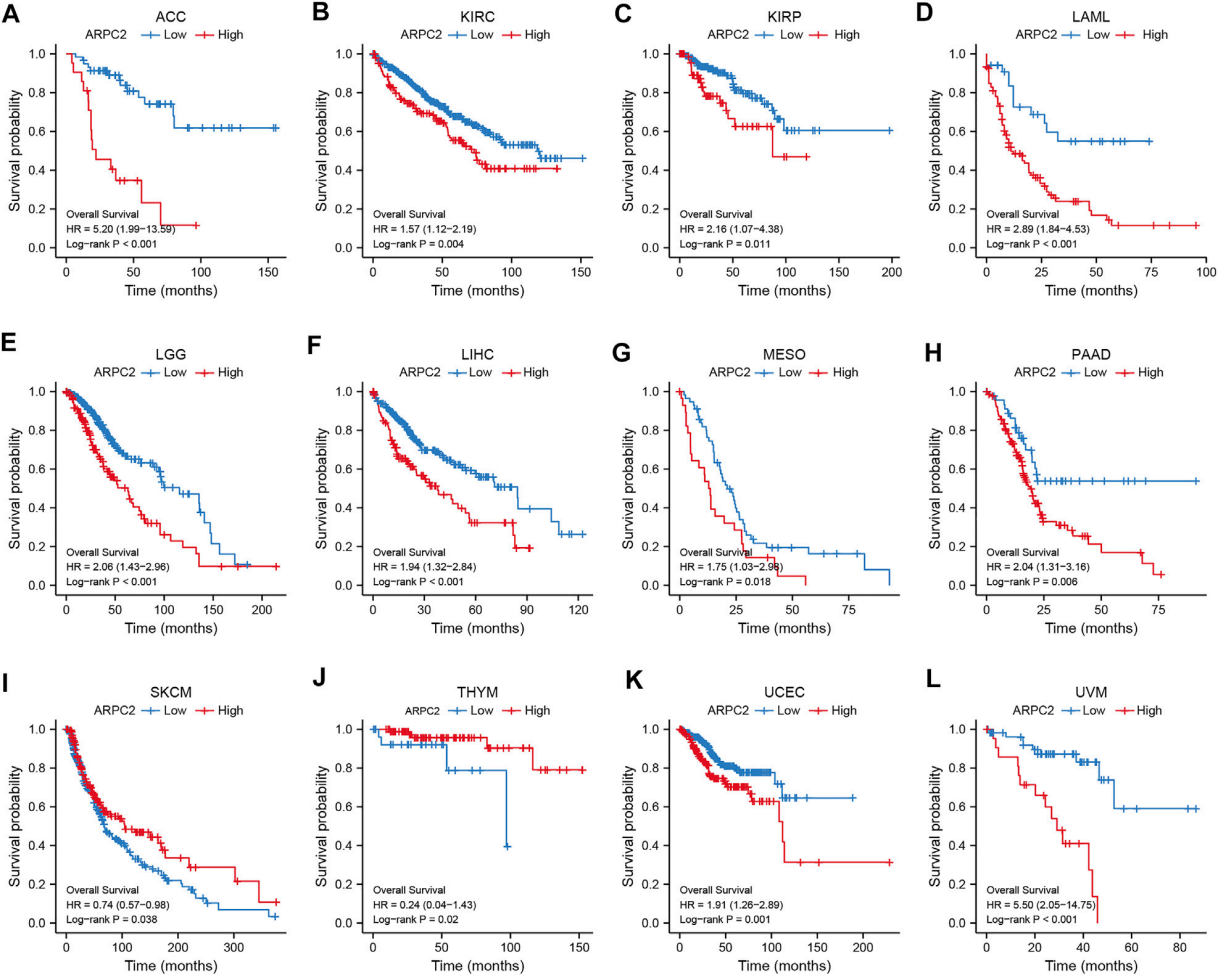
Significant association of ARPC2 expression and overall survival in 12 types of cancer based on Kaplan–Meier analysis. **(A)** Correlation in ACC. **(B)** Correlation in KIRC. **(C)** Correlation in KIRP. **(D)** Correlation in LAML. **(E)** Correlation in LGG. **(F)** Correlation in LIHC. **(G)** Correlation in MESO. **(H)** Correlation in PAAD. **(I)** Correlation in SKCM. **(J)** Correlation in THYM. **(K)** Correlation in UCEC. **(L)** Correlation in UVM.

**FIGURE 4:**
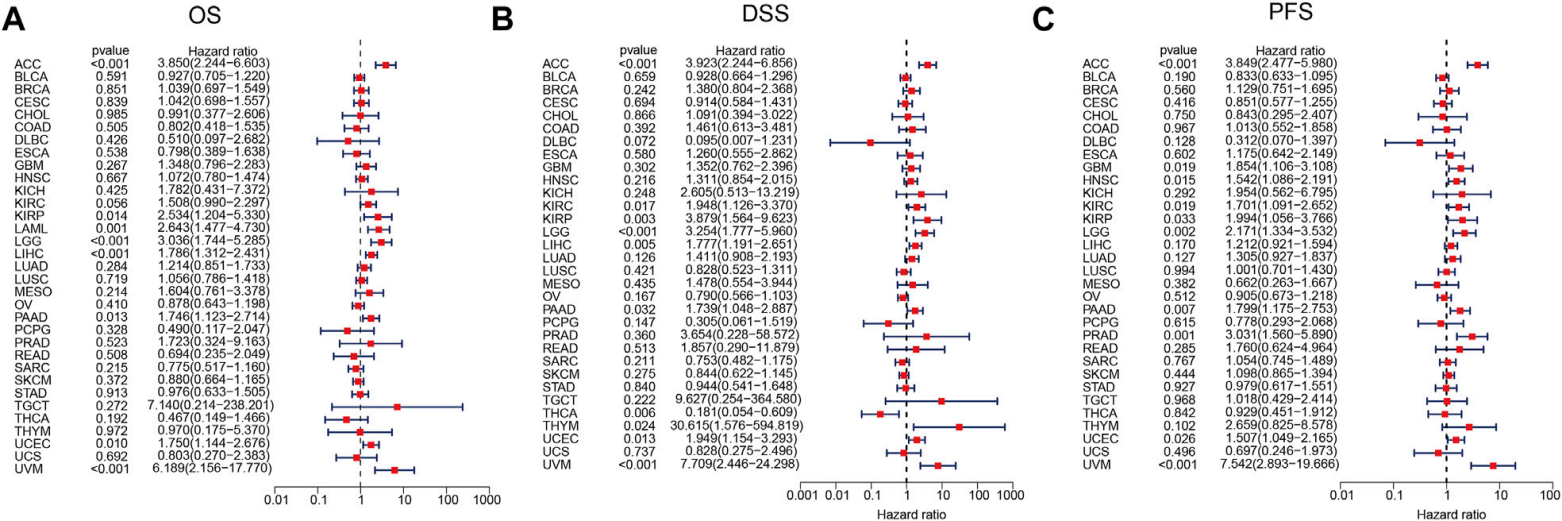
Association of ARPC2 expression and pan-cancer OS, DSS, and PFI based on univariate Cox regression analysis. **(A)** Correlation between ARPC2 expression and OS. **(B)** Correlation between ARPC2 expression and DSS. **(C)** Correlation between ARPC2 expression and PFI. OS, overall survival; DSS, disease-specific survival; and PFI, progression-free interval.

Kaplan–Meier survival and univariate Cox regression analyses were performed to explore the pan-cancer prognostic value of ARPC2, for determining the outcomes OS, DSS, and PFI. The Kaplan–Meier analysis showed that high ARPC2 expression was associated with worse OS in patients with ACC, KIRC, KIRP, LAML, LGG, LIHC, MESO, PAAD, UCEC, and UVM, whereas the opposite results were observed in patients with SKCM and THYM (Figure 3). Upregulated ARPC2 expression was negatively linked with the DSS of patients with ACC, HNSC, KIRC, KIRP, LGG, LIHC, LUAD, MESO, PAAD, UCEC, and UVM (Supplementary Figure S1). The PFI results indicated that an elevated ARPC2 expression predicted worse PFI in patients with ACC, HNSC, GBM, KIRC, KIRP, LGG, PAAD, PRAD, UCEC, and UVM (Supplementary Figure S2). Therefore, we concluded that higher expression levels of APRC2 in ACC, KIRC, KIRP, LGG, PAAD, UCEC, and UVM were associated with worse OS, PFI, and DSS. In addition, the univariate Cox regression analyses indicated that high ARPC2 expression was a risk factor for OS in patients with ACC, KIRP, LAML, LGG, LIHC, PAAD, UCEC, and UVM (Figure 4A). The univariate analysis of DSS showed that poor DSS was strongly correlated with high APRC2 expression in patients with ACC, KIRC, KIRP, LGG, LIHC, LUAD, MESO, PAAD, UCEC, and UVM (Figure 4B). The PFI results of the univariate Cox regression analysis were consistent with those of the Kaplan–Meier survival analysis (Figure 4C). Collectively, the Kaplan–Meier survival and univariate Cox regression analyses showed that the high expression of ARPC2 in ACC, KIRP, LAML, LGG, LIHC, PAAD, UCEC, and UVM predicted worse OS. The aforementioned results demonstrate the potential of ARPC2 as a prospective pan-cancer prognostic biomarker, especially for ACC, HNSC, KIRC, KIRP, LGG, LIHC, MESO, PAAD, UCEC, and UVM.

### Correlation Analysis of APRC2 With Clinicopathological Features in Multiple Cancer Types

**FIGURE 5:**
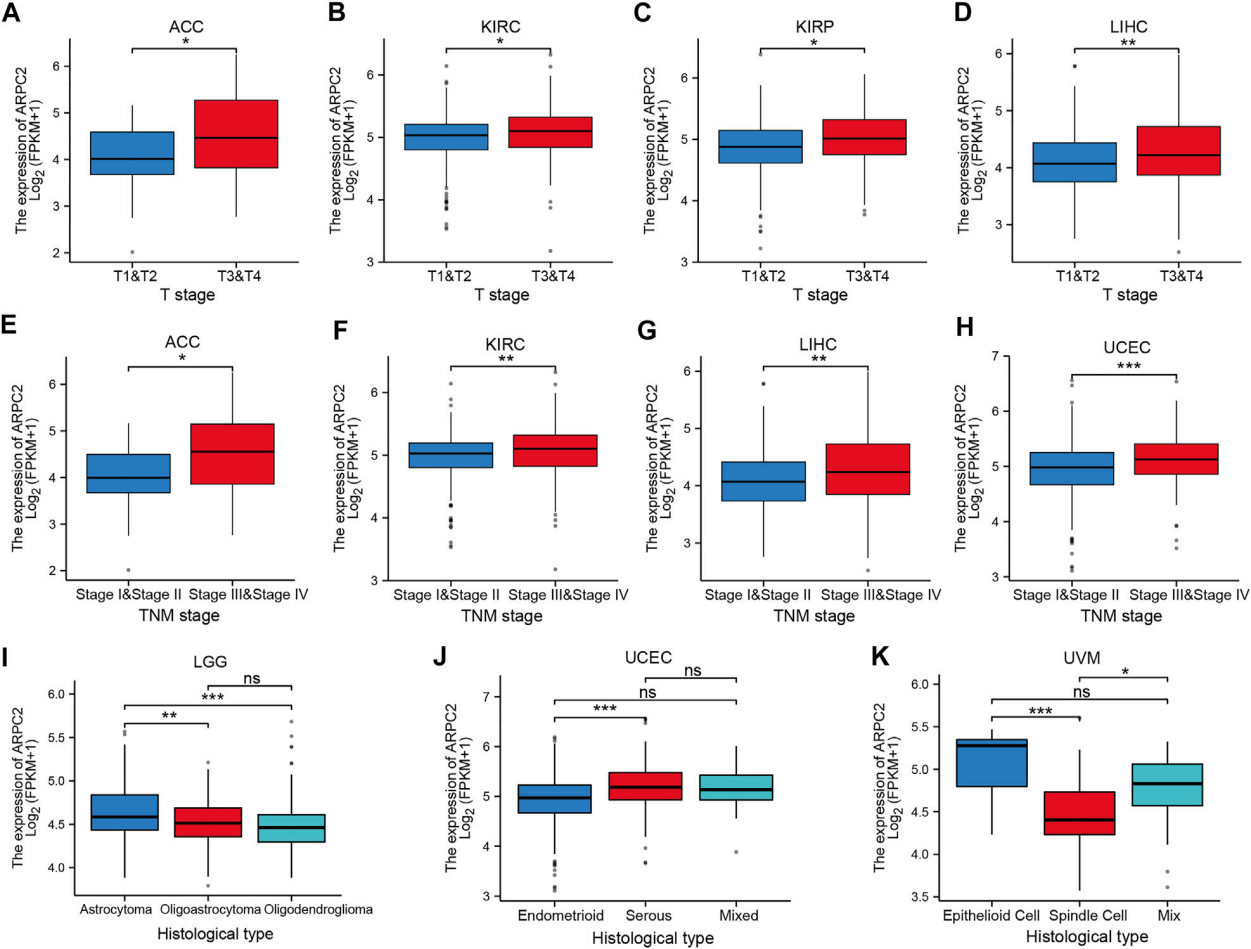
Correlation between ARPC2 expression and clinicopathologic features in various cancers (the correlations with *p* < 0.05 are displayed). **(A–D)** Correlation between ARPC2 expression and the clinical T stage in ACC, KIRC, KIRP, and LIHC. **(E–H)** Correlation between ARPC2 expression and the pathologic TNM stage in ACC, KIRC, LIHC, and UCEC. **(I–K)** Correlation between ARPC2 expression and histological type in LGG, UCEC, and UVM. ns, no significance; *p < 0.05; **p < 0.01; and ***p < 0.001.

To further elucidate the potential impact of ARPC2 on pan-cancer prognosis, the association between ARPC2 and clinicopathological characteristics was evaluated in 10 cancer types (ACC, HNSC, KIRC, KIRP, LGG, LIHC, MESO, PAAD, UCEC, and UVM), whose prognoses were significantly affected by the expression of ARPC2 based on the previously mentioned Kaplan–Meier analyses. Considering that the clinical tumor stage (T stage), pathological TNM stage, and histological type were prognostic factors for most cancers, we compared the ARPC2 expression levels in patients with different stages and histological types. As shown in Figures 5A–K, patients with more advanced clinical T stages displayed higher mRNA expression of ARPC2 in ACC, KIRC, KIRP, and LIHC. The ARPC2 expression levels in ACC, KIRC, LIHC, and UCEC were higher in patients with higher pathological TNM stages (*p* < 0.05). In addition, ARPC2 expression levels were related to the histological type in LGG, UCEC, and UVM. However, there was no significant correlation between ARPC2 expression and the T stage, TNM stage, and histological type in other cancer types (HNSC, MESO, and PAAD). Overall, it can be concluded that ARPC2 may promote tumor progression, thereby contributing to the poor prognosis of ACC, KIRC, KIRP, LIHC, and UCEC.

### Relevance of ARPC2 Expression to the TME and Immune Infiltration Levels in Pan-Cancer

**FIGURE 6:**
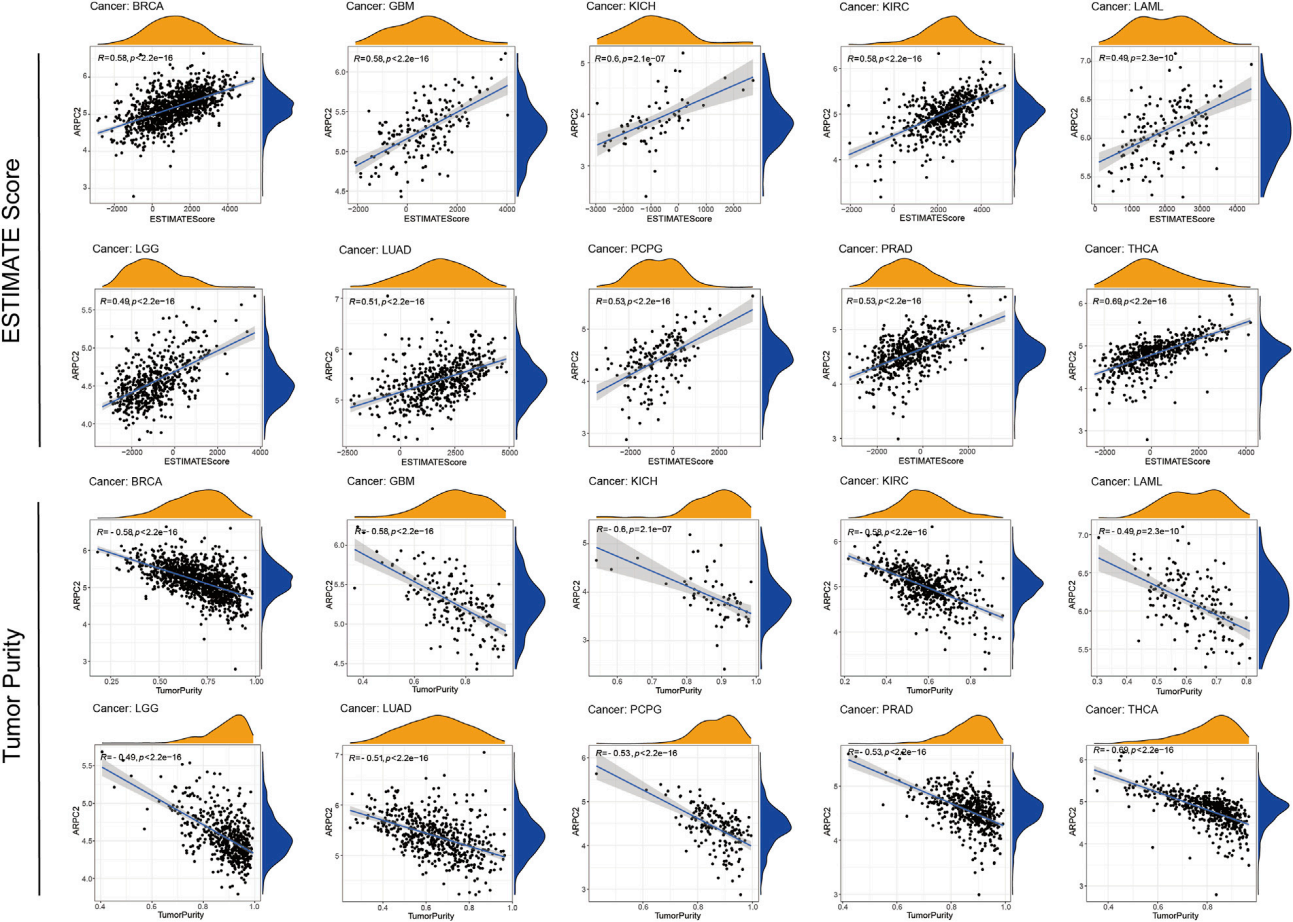
Correlation between ARPC2 expression and the ESTIMATE score and tumor purity of the tumor microenvironment in pan-cancer. The top 10 cancers with most significant association were displayed.

The TME plays a crucial role in tumorigenesis and progression, in which immune and stromal cells are two major non-tumor components that are important for tumor prognosis (Kurebayashi et al., 2018). In the aforementioned analyses, we found relatively higher expression of ARPC2 in immune cells. Thus, we further examined the correlation of ARPC2 with the TME and immune cell infiltration to examine the role of ARPC2 in tumor immunity. Spearman’s correlation analysis, as highlighted in Figure 6; Supplementary Figure S3, showed that ARPC2 expression was positively correlated with the immune, stromal, and ESTIMATE scores in most tumor types. A negative association was observed between ARPC2 and tumor purity, indicating that ARPC2 upregulation in cancer tissues may promote the infiltration of immune and stromal cells in the TME. The top 10 cancer types with the most significant association between ARPC2 expression and TME scores were BRCA, GBM, KICH, KIRC, LAML, LGG, LUAD, PCPG, PRAD, and THCA.

**FIGURE 7:**
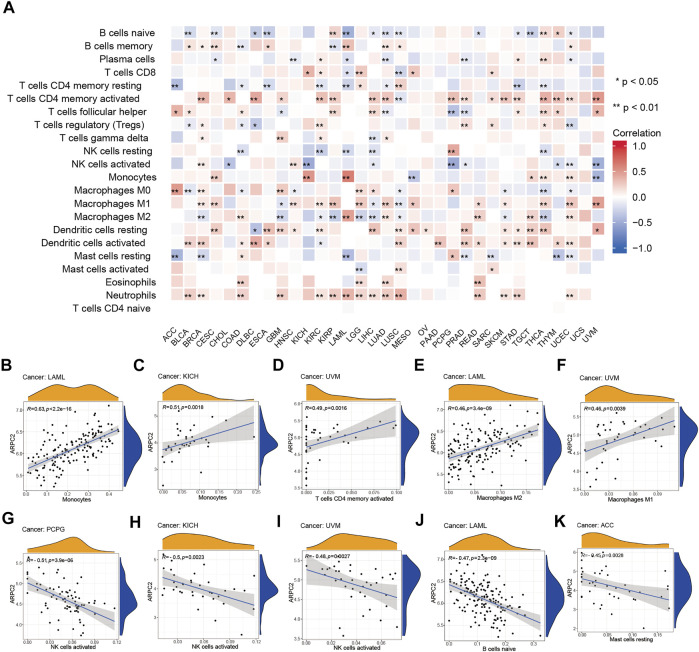
Correlation between ARPC2 and immune infiltration cells in pan-cancer based on the CIBERSORT algorithm. **(A)** Correlation between ARPC2 expression and the proportion of 22 immune infiltration cells. **(B–F)** Top five cancers with most significant positive association between ARPC2 expression and immune cells were displayed. **(G–K)** Top five cancers with most significant negative association between ARPC2 and immune cells are displayed. *p < 0.05; **p < 0.01; and ***p < 0.001.

Next, we performed an in-depth exploration to determine which classes of immune cells are associated with ARPC2 expression. As shown in Figure 7A, ARPC2 expression was most relevant to immune infiltration levels in UCEC. The results of the correlation analysis suggested that ARPC2 was positively correlated with neutrophils, activated dendritic cells, M1 macrophages, M2 macrophages, monocytes, activated memory CD4 T cells, and B memory cells and negatively associated with resting mast cells, M0 macrophages, activated NK cells, regulatory T cells (Tregs), plasma cells, and naïve B cells. The activated memory CD4 T cells, activated dendritic cells, resting dendritic cells, M1 macrophages, and neutrophils were positively associated with ARPC2 in most tumors, whereas naïve CD4 T cells did not correlate with ARPC2 expression in 33 cancer types. The top five tumor types whose immune cells were most positively or negatively correlated to ARPC2 expression were present in scatterplots (Figures 7B–K). The results showed that ARPC2 had significantly positive correlations with monocytes in LAML and KICH, activated memory CD4 T cells, and M1 macrophages in UVM and M2 macrophages in LAML. However, ARPC2 was inversely correlated with activated NK cells in PCPG, KICH, and UVM; the naïve B cells in LAML and resting mast cells in ACC were negatively correlated with ARPC2 expression.

### ARPC2 Predicting Treatment Response to ICIs

**FIGURE 8:**
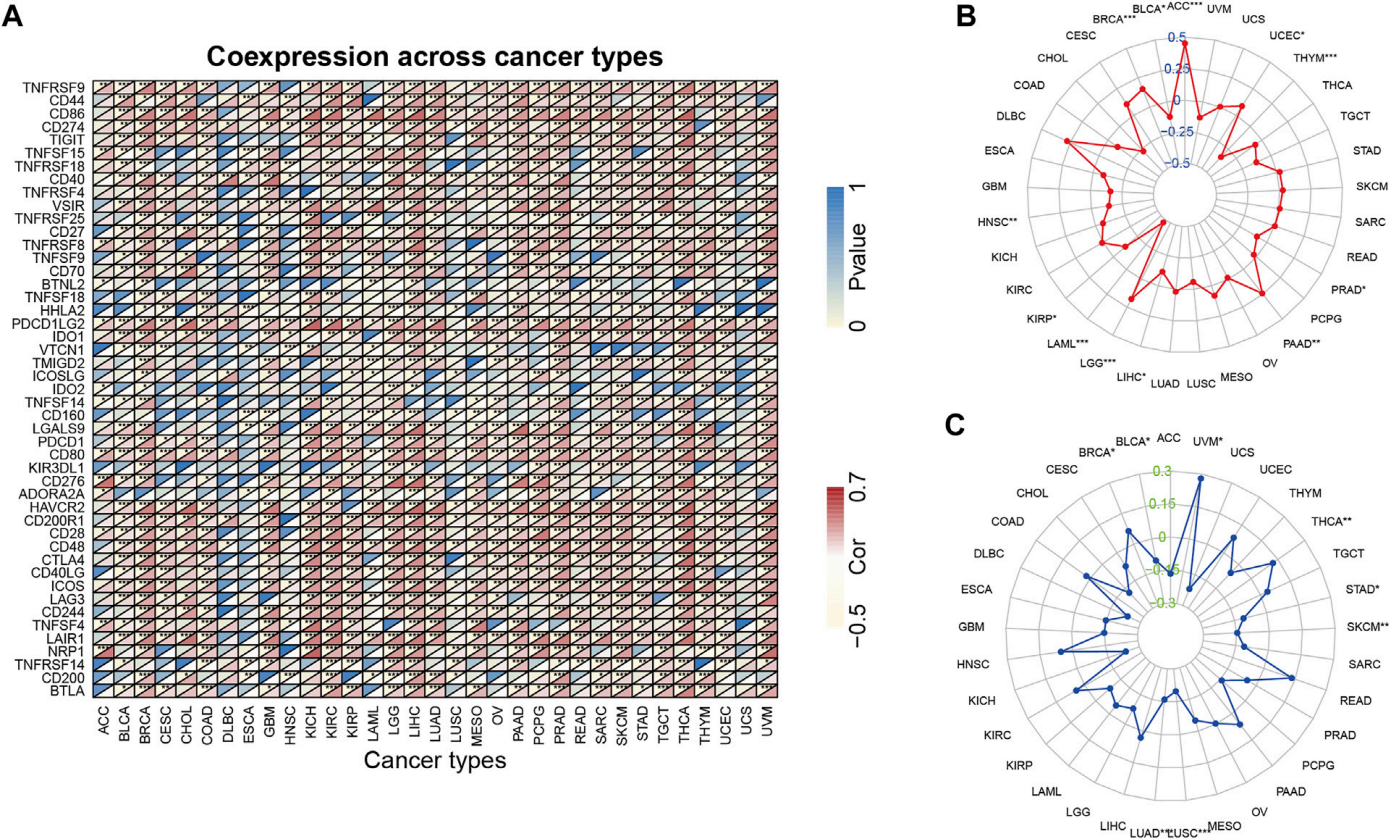
Relationship between ARPC2 expression and pan-cancer immune checkpoint inhibitors therapy. **(A)** Relationship between ARPC2 expression and 47 immune checkpoint-related genes. **(B)** Relationship between ARPC2 expression and tumor mutational burden (TMB). **(C)** Correlation between ARPC2 expression and microsatellite instability (MSI). *p < 0.05; **p < 0.01; and ***p < 0.001.

Immune checkpoint-related genes, such as PD-1 (PDCD1), PD-L1 (CD274), PD-L2 (PDCD1LG2), and CTLA4, have been reported to be the effective predictors of ICIs. In this study, we first analyzed the relationship between ARPC2 expression and 47 immune checkpoint-related genes using expression data from TCGA in 33 cancer types. The results indicated that ARPC2 was significantly correlated with immune-related genes in most cancers (Figure 8A). For example, ARPC2 was positively correlated with 45 of 47 genes, except for ADORA2A and TNFSF14 in HCC. However, fewer associations between ARPC2 and immune genes were observed in DLBC, ESCA, HNSC, and UCS samples. TMB and MSI were closely correlated with the effects of immune checkpoint therapy. Therefore, we further explored the relevance of ARPC2 expression to TMB and MSI in pan-cancer. The results showed that the expression level of ARPC2 was positively correlated with TMB in ACC, BRCA, UCEC, PAAD, and LGG, and the strongest association with TMB was found in ACC (R = 0.45, *p* = 3.10E-05), based on Spearman’s coefficient correlation. In contrast, negative correlations were observed for BLCA, HNSC, KIRP, LAML, LIHC, and THYM (Figure 8B). The expression of ARPC2 was positively correlated with MSI in BRCA, THCA, and UVM, while negatively correlated with MSI in BLCA, LUAD, LUSC, SKCM, and STAD (Figure 8C). These results suggest that ARPC2 can be used to predict the response to ICIs in certain cancers.

### ARPC2 Expression Is Upregulated in HCC Cell Lines and HCC Tissues

**FIGURE 9:**
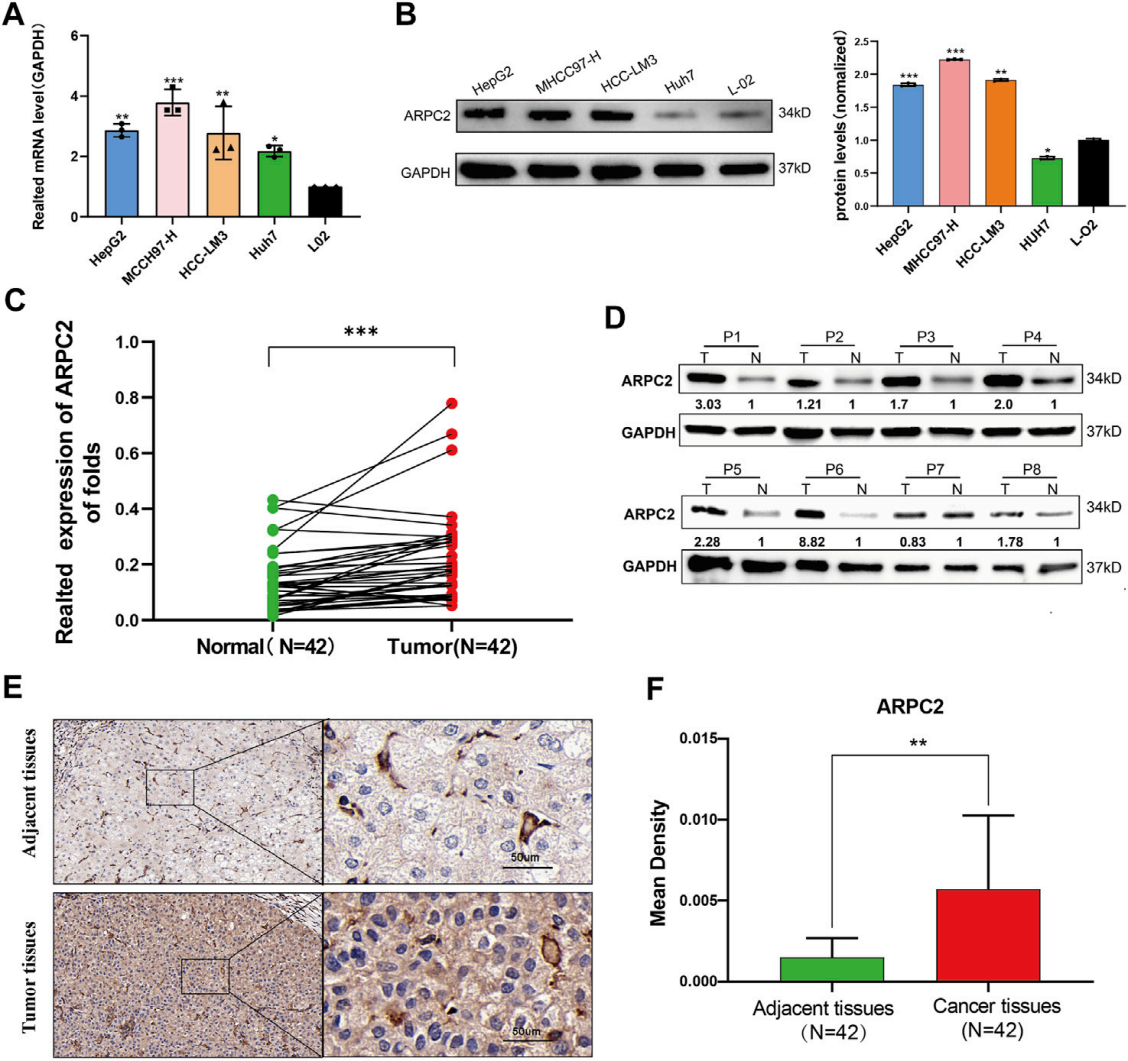
Differential expression of APRC2 in HCC cell lines and human primary HCC tissues. **(A)** RT-qPCR analysis of ARPC2 mRNA expression in normal liver cell line (L-02), and four HCC cell lines (HCC-LM3, MHCC97-H, HepG2, and Huh-7). Error bars represent the SD of triplicate experiments. **(B)** Western blot analysis of ARPC2 protein expression in four HCC cell lines and normal liver cell line. Error bars represent the SD of triplicate measurements. **(C)** mRNA expression of ARPC2 in 42 pairs HCC tissues and adjacent para-carcinoma tissues was evaluated by RT-qPCR. GAPDH was used as an internal control. **(D)** Western blot analysis of ARPC2 protein expression in eight randomly picked HCC tumor tissues and the corresponding adjacent normal tissues. The values were presented as relative protein expression levels of ARPC2. **(E)** Representative images of ARPC2 expression in the HCC tissues and adjacent normal liver tissues, analyzed by immunohistochemistry, original magnifications: ×40 and ×200. Scale bars: 50 μm. **(F)** Quantitative analysis of ARPC2 expression in HCC tissues based on mean density of immunohistochemical staining. Error bars represent the SD of multiple tissues. *p < 0.05; **p < 0.01; and ***p < 0.001.

Our pan-cancer bioinformatics analyses showed that ARPC2 was significantly overexpressed in HCC tissues and was closely associated with OS and DSS in patients with HCC. Moreover, we found a significant correlation between ARPC2 expression and tumor immunity in HCC. Therefore, we focused on exploring the biological functions of ARPC2 in HCC. We first examined the differential expression in HCC cell lines (including HCC-LM3, MHCC97-H, HepG2, and Huh-7) using RT-qPCR and western blot analysis. The results suggested that the mRNA expression of ARPC2 was significantly upregulated in the four HCC cell lines compared with that in the normal liver cell line L-02 (Figure 9A). Similarly, the protein expression level of ARPC2 was significantly higher in HCC-LM3, MHCC97-H, and HepG2 cells than in normal liver cells (Figure 9B). Among the four HCC cell lines, the expression of ARPC2 was relatively high in HCC-LM3 and MHCC97-H cells, but was relatively low in HepG2 and Huh-7 cells. Forty two paired HCC tissues and adjacent normal tissues were collected from HCC patients after hepatectomy, and the clinical and pathological features of the HCC patients are shown in Table 2. RT-qPCR, western blotting, and immunohistochemical staining were used to determine the expression of ARPC2 in tissue samples. The results of RT-qPCR suggested that the expression of ARPC2 was higher in HCC tissues than that in adjacent para-carcinoma tissues (Figure 9C), which was consistent with TCGA results. Eight patients were randomly selected for western blot analysis, and the results indicated that ARPC2 expression was upregulated in HCC tumor tissues compared with that in adjacent normal liver tissues in seven of those samples (Figure 9D). In addition, immunohistochemistry assays for ARPC2 were performed on samples from 42 patients with HCC, and the mean density was used for quantitative analysis of gene expression in HCC tissues. The results showed increased staining in HCC tissues, and the mean density value of ARPC2 immunohistochemical staining of cancer tissues was higher than that of para-carcinoma tissues (Figures 9E,F), which is consistent with the results of western blotting.

**TABLE 2:** Clinical and pathological features of HCC patients.

Characteristics	Number of cases (%)
Age	
≤60	27 (64.3)
>60	15 (35.7)
Gender	
Male	38 (90.5)
Female	4 (9.5)
HBsAg	
Negative	10 (23.8)
Positive	32 (76.2)
Child–Pugh classification	
A	21 (50)
B	21 (50)
AFP	
≤400 ng/ml	25 (59.5)
>400 ng/ml	17 (40.5)
Liver cirrhosis	
Absent	9 (21.4)
Present	33 (78.6)
Tumor number	
Single	32 (76.2)
Multiple	10 (23.8)
Lymph nodes metastasis	
N0	39 (92.9)
N1	3 (7.1)
Distant metastasis	
M0	42 (100)
M1	0 (0)
Edmondson–Steiner grades	
I	3 (7.1)
II	21 (50)
III	18 (42.9)
IV	0 (0)

### ARPC2 Silencing Inhibits Proliferation, Migration, and Invasion, While Accelerating HCC Cell Apoptosis

**FIGURE 10:**
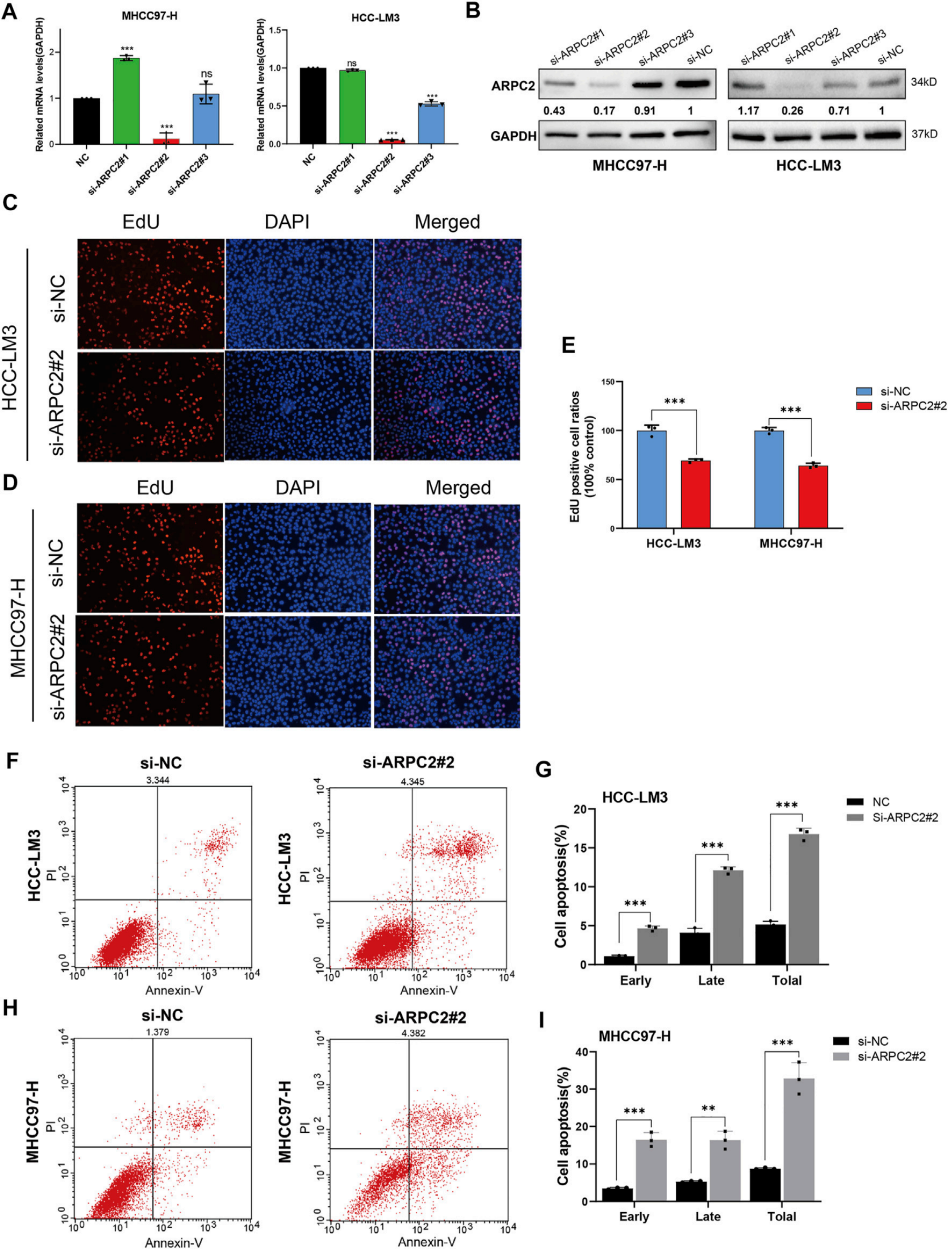
Downregulation of ARPC2 reduces proliferation and promotes apoptosis in HCC cells. **(A,B)** Inhibiting efficiency of different siRNA–ARPC2 was evaluated in HCC-LM3 and MHCC97-H cells with RT-qPCR and western blotting, respectively. Error bars represent the SD of triplicate experiments. The values of western blot presented relative protein expression levels of ARPC2. **(C–E)** HCC-LM3 and MHCC97-H cells were transfected with siRNA–ARPC2#2 or negative control (si-NC) for 48 h, and the proliferative ability was assessed using EdU assays. Representative images **(C,D)** and the number of proliferative cells are shown **(E)**. Original magnification is ×200. Error bars represent the SD of triplicate measurements. **(F–I)** Annexin V assays followed by flow cytometry was used to evaluate the apoptosis rate in HCC cells treated with si-ARPC2 or si-NC, including early apoptosis, late apoptosis, and total apoptosis. Error bars of the histogram represented the SD of triplicate experiments. *p < 0.05; **p < 0.01; and ***p < 0.001.

In our previous study, we reported that ARPC2 expression mainly affected cell apoptosis, cell cycle, and the MAPK and WNT signaling pathways in HCC, based on KEGG enrichment analysis (Huang et al., 2021). Therefore, we explored the underlying impact of ARPC2 on the proliferation and metastasis of HCC cells. Given that ARPC2 expression was relatively higher in HCC-LM3 and MHCC97-H cells, we selected the two cell lines for transfection with siRNA-targeting ARPC2. First, the interference efficiency of three siRNA fragments (si-ARPC2#1, si-ARPC2#2, and si-ARPC2#3) was verified by qPCR and western blotting. The results showed that si-ARPC2#2 could effectively silence ARPC2, and the knockdown efficiency was >70% in HCC cells (Figures 10A,B). Thus, we used si-ARPC2#2 for further experiments. Next, we explored the role of ARPC2 in HCC cell proliferation and apoptosis using EdU staining assays and flow cytometry. The results of the EdU assays showed that the knockdown of ARPC2 significantly reduced the proliferative capacity of HCC cells compared to controls (Figures 10C–E). Annexin V assays followed by flow cytometry showed that the downregulation of ARPC2 markedly increased cell apoptosis compared with control cells, including early apoptosis and late apoptosis (Figures 10F,G). In general, ARPC2 affected the proliferation and apoptosis of HCC cells.

**FIGURE 11:**
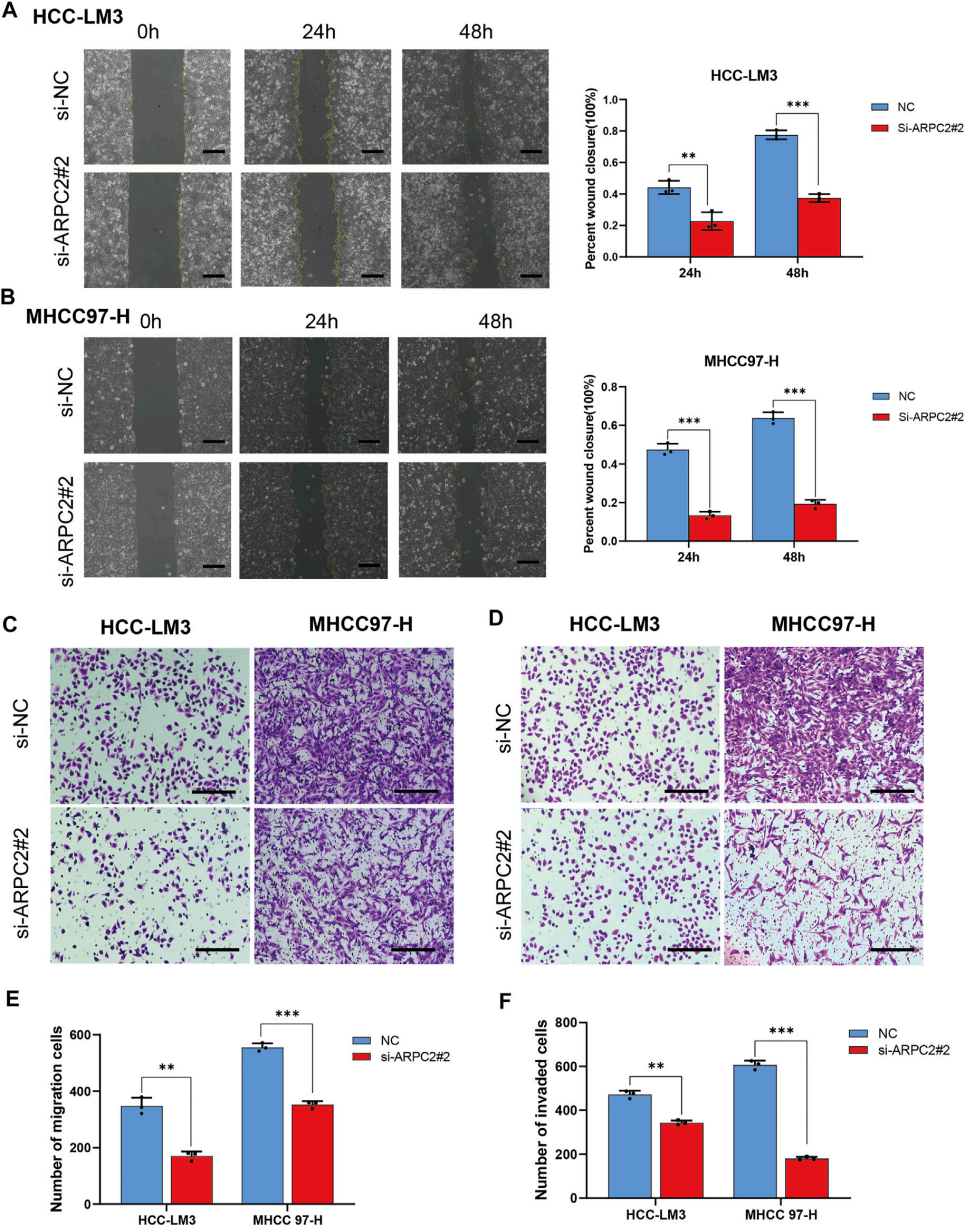
Silencing ARPC2 inhibits the migration and invasion in HCC cells. **(A,B)** Migration ability was assessed by scratch assay, representative images (left) are shown (original magnification, ×100; scale bars, 200 μm), and wound healing area are calculated (right). Error bars of the histogram represented the SD of triplicate measurements. **(C–E)** Transwell assay without Matrigel was used to assess the migration ability of the transfected HCC cells; **(C)** representative images are shown (original magnification, ×200; scale bars, 200 μm); **(E)** histogram showed the number of migration cells. Error bars of the histogram represented the SD of triplicate measurements. **(D–F)** Transwell assay with Matrigel was applied to evaluate the invasion ability; **(D)** representative images are shown (original magnification, ×200; scale bars, 200 μm); **(F)** histogram showed the number of invasion cells. Error bars of the histogram represented the SD of triplicate measurements. *p < 0.05; **p < 0.01; and ***p < 0.001.

The potential role of APRC2 in the migration and invasion of HCC cells was verified using scratch and Transwell assays. We found that compared with the control HCC cells, the migration rate was significantly reduced in the ARPC2-silenced cells after 24 and 48 h of scratching (Figures 11A,B). The same results were observed in Transwell assays without Matrigel, HCC-LM3 and MHCC97-H cells transfected with siRNA specific to ARPC2 exhibited significantly weaker migration capacity than the control groups (Figures 11C–E). In addition, the results of the Transwell invasion assays demonstrated that the cell invasion ability of the ARPC2 knockdown groups was significantly reduced compared with that of the control groups (Figures 11D–F).

### The Overexpression of ARPC2 Promotes HCC Cell Proliferation, Migration, and Invasion

**FIGURE 12:**
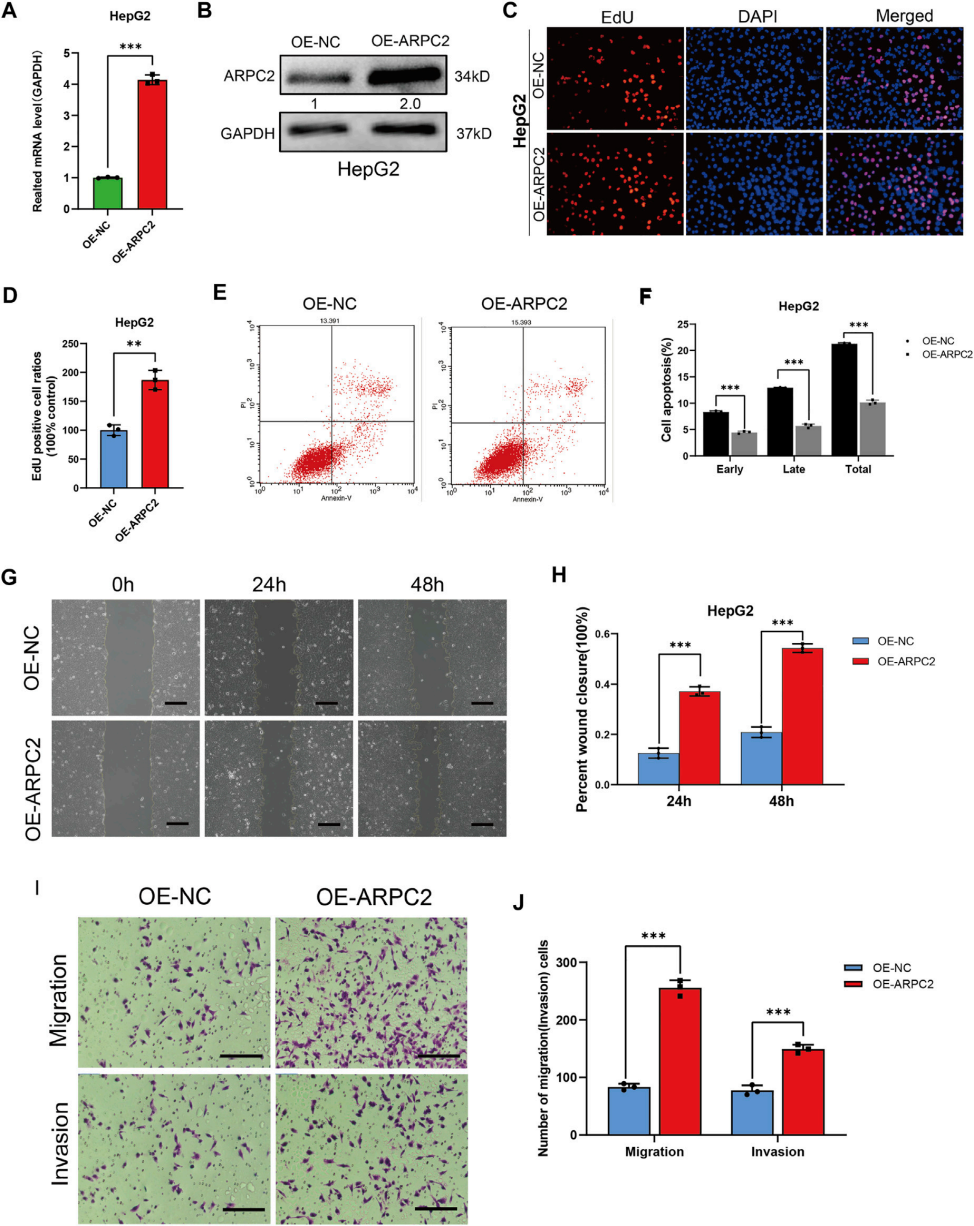
ARPC2 overexpression promotes cells proliferation, migration, invasion, and reduces cells apoptosis in HCC. **(A)** Overexpression efficiency was detected in the HepG2 cell with qPCR. Error bars represent the SD of triplicate experiments. **(B)** Overexpression efficiency was detected in HepG2 cells with western blotting. The values in picture presented relative protein expression levels of ARPC2. **(C,D)** HepG2 cell was transfected with OE-ARPC2 or negative control (OE-NC) for 48 h, and the proliferative ability was assessed by EdU assays; **(C)** representative images, original magnification, ×200; **(D)** Number of proliferative cells are calculated. Error bars represent the SD of triplicate measurements. **(E,F)** Annexin V assays followed by flow cytometry was used to evaluate the apoptosis rate of HepG2 transfected with overexpression plasmids; representative images **(E)**, and the statistical plot of the number of apoptosis cells including early apoptosis, late apoptosis, and total apoptosis **(F)**. Error bars of the histogram represented the SD of triplicate experiments. **(G,H)** Migration ability was assessed in the HepG2 cell transfected with OE-ARPC2 or negative control (OE-NC) using scratch assay, representative images **(G)** are shown in oh, 24 h and 48 h after scratching (original magnification, ×100; scale bars, 200 μm), and the wound healing area are calculated **(H)**. Error bars of the histogram represented the SD of triplicate measurements. **(I,J)** Transwell assays were performed to evaluate the migration and invasion ability, representative images are shown **(I)** (original magnification, ×200; scale bars, 200 μm), the histogram showed the number of migration or invasion cells. Error bars of the histogram represented the SD of triplicate measurements. *p < 0.05; **p < 0.01; and ***p < 0.001.

To further investigate the role of ARPC2 in regulating HCC cellular behaviors, EdU assays, scratch assays, Transwell assays, and flow cytometry analysis of cell apoptosis were conducted in HepG2 cells transfected with ARPC2 overexpression plasmids. The mRNA and protein expression levels of ARPC2 were elevated in the OE-ARPC2 group than in the OE-NC group (Figures 12A,B). Regarding cell proliferation and apoptosis, the EdU assay results indicated that ARPC2 overexpression in HepG2 cells promoted cell proliferation (*p* < 0.01) (Figures 12C,D). The flow cytometry analysis of cell apoptosis showed that the number of apoptotic cells in the overexpression group was significantly reduced compared with that in the control group, including early, late, and total apoptosis (*p* < 0.001) (Figures 12E,F). In addition, ARPC2 overexpression significantly enhanced cell migration and invasion in HepG2 cells, as demonstrated by scratch (Figures 12G,H) and Transwell assays (Figures 12I,J).

## Discussion

The Arp2/3 protein complex consists of seven subunits (ACTR2, ACTR3, and ARPC1-5) and is involved in actin filament nucleation and depolymerization processes, which are crucial for lamellipodia formation, phagocytosis, cell adhesion, and cell motility (Kiuchi et al., 2011). The deregulation of Arp2/3 is significantly correlated with malignant phenotypes and poor prognosis in various cancers (Molinie and Gautreau, 2018). However, these studies were focused on a single cancer type with limited samples; the expression patterns, prognostic value, and biological significance of ARPC2 in most types of cancer remain largely unknown. In this study, we conducted a comprehensive bioinformatics analysis of ARPC2 in 33 cancer types based on multiple databases, including gene expression analysis in different tissues and cell lines, gene mutation, DNA methylation, prognostic value and its association with clinical features, TME, tumor immune infiltration cells, and response to ICIs. Our results provide insights into the expression patterns and clinical significance of ARPC2 pan-cancer and into the relationship among ARPC2 expression, TME, and tumor immunity. This study revealed the oncogenic function of ARPC2 in HCC using a series of experiments for the first time.

Previous studies have reported that ARPC2 is overexpressed in various tumor tissues and is closely associated with prognosis. For example, ARPC2 is more highly expressed in gastric cancer tissues than in normal gastric tissues, and ARPC2-positive cases are associated with poor relapse-free survival and OS and significantly associated with a large tumor size, lymph node invasion, and high tumor stage in gastric cancer (Zhang J et al., 2017). The expression level of ARPC2 is higher in breast cancerous tissues than in adjacent noncancerous tissues, and ARPC2 is highly associated with the tumor stage and nodal metastasis in patients with breast cancer (Cheng et al., 2019). Gene-based markers as biological signatures can effectively predict tumor outcomes. Song et al. (2015) developed a systematic approach to uncover novel survival-related subnetwork signatures that could uncover biological mechanisms in metastatic breast cancer. (In our study, we developed a new molecular biomarker for predicting the survival outcomes of multiple cancer types based on single-gene expression data. First, we systematically examined the ARPC2 expression patterns of 33 human cancer types according to TCGA and GTEx databases. ARPC2 was overexpressed in most tumor tissues, including BRCA, CHOL, ESCA, GBM, HNSC, KIRC, KIRP, LAML, LIHC (HCC), LGG, OV, PAAD, STAD, THCA, UCEC, and UVM. This result is in line with previous findings on BRCA and STAD. Moreover, patients with a higher level of ARPC2 exhibited markedly poorer OS when suffering from HNSC, KIRC, KIRP, LIHC, LGG, MESO, PAAD, UCEC, and UVM, compared with those with a low level of ARPC2. Conversely, the ARPC2 expression levels decreased in ACC, KICH, LUAD, PRAD, SKCM, and THYM tissues, and a low ARPC2 expression was significantly associated with shorter OS in SKCM and THYM. Interestingly, the high expression of ARPC2 was associated with worse OS, PFI, and DSS in ACC. This contradiction in ACC might be due to the limited sample size. To determine the effect of ARPC2 expression on ACC, HNSC, KIRC, KIRP, LIHC, LGG, MESO, PAAD, UCEC, and UVM prognosis, we examined the association between ARPC2 expression and clinical features. The results showed a close correlation between ARPC2 expression and the tumor stage in ACC, KIRC, KIRP, LIHC, and UCEC; the higher the ARPC2 expression levels, the higher the T or TNM stage. In addition, the mRNA and protein expression levels of ARPC2 in HCC tissues and cell lines were further verified by qPCR, western blotting, and immunohistochemistry. The results showed that ARPC2 expression was higher in HCC tissues and cell lines than in adjacent normal tissues and normal liver cell lines. In short, these findings suggest that ARPC2 may have distinct prognostic roles in different cancer types and that ARPC2 expression could be a promising prognostic marker in patients with ACC, HNSC, KIRC, KIRP, LIHC, LGG, MESO, PAAD, UCEC, and UVM.

Aberrant gene expression is a common characteristic of cancer cells and is closely associated with the abnormal growth of cells, connecting tumorigenesis, and tumor progression. Gene mutations and DNA methylation can lead to genomic instability, which, in turn, may trigger cancer development (Li et al., 2010). In our study, we found that gene mutations and the methylation of ARPC2 had a vital impact on ARPC2 expression. The main mutation forms of ARPC2 in tumors were “mutation” and “deep deletion,” and the mutations were primarily focused on CESC, UCEC, and SARC. We also discovered, in most cancer types, the ARPC2 mRNA expression was significantly correlated with the CNV percentage, which was mainly reflected in HNSC, BRCA, and BLCA. TMB was positively correlated with ARPC2 expression in ACC, BRCA, LGG, and PAAD. Moreover, we found that the methylation level of ARPC2 was negatively associated with gene expression in 27 tumors, such as THCA, BRCA, and PRAD. In addition, ARPC2 expression was positively correlated with MMR-related genes and DNA methyltransferases in most cancer types, indicating that MMRs and DNA methylation might influence the expression of ARPC2. These findings revealed the potential cause of abnormal ARPC2 expression in cancers from the perspective of tumor mutation and DNA modification.

The Arp2/3 complex plays a vital role in tumor invasion and metastasis. The silencing of the Arp2/3 complex subunits typically reduces the cell migration capacity in pancreatic cancer, bladder cancer, and head and neck squamous cell carcinoma (Kinoshita et al., 2012; Rauhala et al., 2013; Xu et al., 2020). As one of the subunits of Arp2/3, ARPC2 also plays a fundamental role in promoting cell shape changes and locomotion. ARPC2 promotes vascular smooth muscle cell migration (Al Ghouleh et al., 2013). ARPC2 increases breast tumor cell proliferation, migration, and invasion and activates the TGF-β pathway to contribute to epithelial-mesenchymal transition (Cheng et al., 2019). Blocking ARPC2 dramatically inhibits the proliferation and invasion of the human gastric cancer cell line, MKN-28 (Zhang J et al., 2017). In this study, we investigated the biological function of ARPC2 in HCC. The results indicated that the proliferative and migratory abilities were attenuated by ARPC2 silencing in HCC cell lines, while the apoptosis process was significantly intensified after transfection with siRNA-targeting ARPC2. In contrast, the ARPC2 overexpression in HepG2 cells remarkably promoted cell proliferation, migration, and invasion, but decreased cell apoptosis. These results provide novel evidence of the oncogenic function of ARPC2 in HCC. Based on the transcriptomic GSEA in our previous study, we found that ARPC2 is mainly involved in cell apoptosis, cell cycle, MAPK signaling pathway, and WNT signaling pathway (Huang et al., 2021). Recently, the progress of multi-omics technology has shed a more favorable light on the integrative analysis of multiple omics data as it can elucidate the molecular mechanisms of carcinogenesis and tumor development. For example, a previous study reported that transcriptomic, proteomic, and metabolomic molecular profile analyses conducted in patients with brain metastasis could identify key pathways and metabolites between the good and poor prognostic subtypes (Su et al., 2020). Thus, multi-omics data integration and further experimental validation are needed to explore the specific molecular mechanisms of ARPC2 in HCC.

The TME plays an essential role in tumor progression, immune escape, and immunotherapy resistance. Previous studies have suggested that the Arp2/3 complex is implicated in numerous cellular processes, from endocytic trafficking to cell–cell and cell–extracellular matrix (ECM) adhesion, thereby influencing immune cell infiltration and the TME (Rotty et al., 2017). Zhang Y et al. (2017) revealed the critical role of the Arp2/3 complex in surface TCR maintenance and T cell homeostasis and found that loss of ARPC2 causes a dramatic decrease in peripheral T cell numbers and compromises T cell homeostasis by disrupting the integrity of the complex. ARPC2 is also a critical driver of integrin-dependent phagocytosis and chemotaxis of macrophages. In addition, in our previous study, we demonstrated that the expression levels of ARPC2 in HCC tissues are associated with a variety of immune infiltration cells (Huang et al., 2021). However, the relationship of ARPC2 with the pan-cancer TME and tumor immunity remains largely unknown. In this study, we used different algorithms to analyze the correlation between ARPC2 expression and TME as well as immune cell infiltration in pan-cancer. We discovered that the infiltration level of stromal cells and immune cells in the TME was positively correlated with ARPC2 expression in pan-cancer, whereas tumor purity showed the opposite results. Furthermore, we found a close association between ARPC2 and different immune cells and pathways, such as neutrophils, activated memory CD4 cells, and activated dendritic cells. Thus, we speculated that the differential enrichment of immune and stromal cells in different cancer types might explain why ARPC2 expression plays a distinct prognostic role.

In recent years, ICIs have revolutionized cancer therapy and led to durable remission in a wide variety of cancer types (Hiam-Galvez et al., 2021). Nevertheless, only a minority of patients with cancer can benefit from a single ICI and prolong their survival (Jardim et al., 2021). Many unresolved issues remain, such as low objective response rate, unique immune-related toxicities, hyper-progression, and lack of promising predictive markers for immunotherapy effectiveness, which severely hinder the generalization of immunotherapy (Hegde and Chen, 2020). Thus, exploring more accurate biomarkers to forecast the responsiveness of ICIs and further screening of dominant patients for ICIs has become one of the main challenges for cancer treatment. Genomic instability, described as high-TMB and high-MSI, provides immunogenic neoantigens for cancer cells, which is critical for the initiation of cancer immunity and generates a more effective anticancer T cell response (Buttner et al., 2019; Goodman et al., 2020). Tumors with higher TMB or MSI are closely correlated with better ICI outcomes, based on previous research data on non-small cell lung cancer (NSCLC), colon cancer, and metastatic bladder cancer (Rizvi et al., 2015; Rosenberg et al., 2016; Samstein et al., 2019; Schrock et al., 2019). PD-L1 expression and IFN-γ signatures are also associated with the response to ICIs (Camidge et al., 2019). However, the expression levels of PD-L1, TMB, and MSI were not significantly correlated in most cancer subtypes. Thus, a combination of these biomarkers may improve the predictive performance of ICIs. This study provides a comprehensive correlation analysis between ARPC2 expression and current biomarkers of ICI response, including immune-related genes, TMB, and MSI. We found that ARPC2 was significantly associated with ICI-related genes in most cancers. We further revealed that ARPC2 expression was positively correlated with TMB and MSI in BRCA, and a reverse relationship was found in BLCA. These findings indicate that ARPC2 can be applied as an effective predictor of ICI efficacy in certain cancer types.

In summary, systematic analyses were performed to explore the expression and prognostic value of ARPC2 in pan-cancers using multiple databases. We found the expression of ARPC2 was upregulated in most cancer types and associated with poor survival prognosis and unfavorable clinicopathological features. ARPC2 expression might act as a promising prognostic marker in multiple cancers, especially for ACC, HNSC, KIRC, KIRP, LIHC(HCC), LGG, MESO, PAAD, UCEC, and UVM. Moreover, ARPC2 was closely related to the TME, tumor immunity, and response to ICIs, which might be a potential therapeutic target for immunotherapy and guided individualized immunotherapy for cancers. In addition, the experimental results highlighted the cancer-promoting effect of ARPC2 in HCC, which provided a preliminary foundation for the development of biomarker-targeting therapies in HCC.

## Data Availability

The original contributions presented in the study are included in the article/Supplementary Material; further inquiries can be directed to the corresponding author.

## Glossary

ACC: Adrenocortical carcinoma
Arp2/3: Actin-related protein 2/3s
ARPC2: Actin-related protein 2/3 complex subunit 2
BLCA: Bladder urothelial carcinoma
BRCA: Breast invasive carcinoma
CESC: Cervical squamous cell carcinoma and endocervical adenocarcinoma
CHOL: Cholangiocarcinoma
CNV: Copy number variations
COAD: Colon adenocarcinoma
DLBC: Lymphoid neoplasm diffuse large B-cell lymphoma
DSS: Disease-specific survival
EdU: 5-Ethynyl-2′-deoxyuridine
ESCA: Esophageal carcinoma
GBM: Glioblastoma multiforme
GTEx: Genotype-tissue expression database
HCC: Hepatocellular carcinoma
HNSC: Head and neck squamous cell carcinoma
ICIs: Immune checkpoint inhibitors
KICH: Kidney chromophobe
KIRC: Kidney renal clear cell carcinoma
KIRP: Kidney renal papillary cell carcinoma
LAML: Acute myeloid leukemia
LGG: Brain lower grade glioma
LIHC: Liver hepatocellular carcinoma
LUAD: Lung adenocarcinoma
LUSC: Lung squamous cell carcinoma
MESO: Mesothelioma
MMRs: Mismatch repairs
MSI: Microsatellite instability
OS: Overall survival
OV: Ovarian serous cystadenocarcinoma
PAAD: Pancreatic adenocarcinoma
PCPG: Pheochromocytoma and paraganglioma
PFI: Progression-free interval
PRAD: Prostate adenocarcinoma
READ: Rectum adenocarcinoma
RT-qPCR: Real-time reverse transcription quantitative polymerase chain reaction
siRNA: Small interfering RNA
SARC: Sarcoma
SKCM: Skin cutaneous melanoma
STAD: Stomach adenocarcinoma
TCGA: The Cancer Genome Atlas database
TGCT: Testicular germ cell tumors
THCA: Thyroid carcinoma
THYM: Thymoma
TMB: Tumor mutational burden
TME: Tumor microenvironment
TNM: Tumor-node-metastasis
UCEC: Uterine corpus endometrial carcinoma
UCS: Uterine carcinosarcoma
UVM: Uveal melanoma
