# Supplementary material for: ARPC2: A Pan-Cancer Prognostic and Immunological Biomarker That Promotes Hepatocellular Carcinoma Cell Proliferation and Invasion
Source: Front Cell Dev Biol. 2022 Jun 6;10:896080. doi: 10.3389/fcell.2022.896080 (PMC9207441; doi:10.3389/fcell.2022.896080)
Supplement: Supplementary file 1 [file Table1.DOCX]

**Table S1. Primers for qRT-PCR analysis and siRNA sequence targeting ARPC2**

| Name | Sequences (5'—3') |
| --- | --- |
| qRT-PCR primers  ARPC2  GAPDH  siRNA sequences  si-APRC2#1  si-ARPC2#2  si-ARPC2#3  si-NC | F: AAAGAGGGTGTACGGGAGTTT; R: TGGATGCCGGAAGATTTTCAAG  F: GGAGCGAGATCCCTCCAAAAT; R: GGCTGTTGTCATACTTCTCATGG  UUUCAAAGAAAUACUGACCTT  UUGAUGCACAAUGGAAUCCTT  UUCUUUGAACUCCUGCAUGTT  ACGUGACACGUUCGGAGAATT |

qRT-PCR: quantitative real-time reverse transcription polymerase chain reaction

siRNA: small interfering RNA

F: forward primer

R: reverse primer
