# Supplementary figures and images for: ARPC2: A Pan-Cancer Prognostic and Immunological Biomarker That Promotes Hepatocellular Carcinoma Cell Proliferation and Invasion
Source: Front Cell Dev Biol. 2022 Jun 6;10:896080. doi: 10.3389/fcell.2022.896080 (PMC9207441; doi:10.3389/fcell.2022.896080)

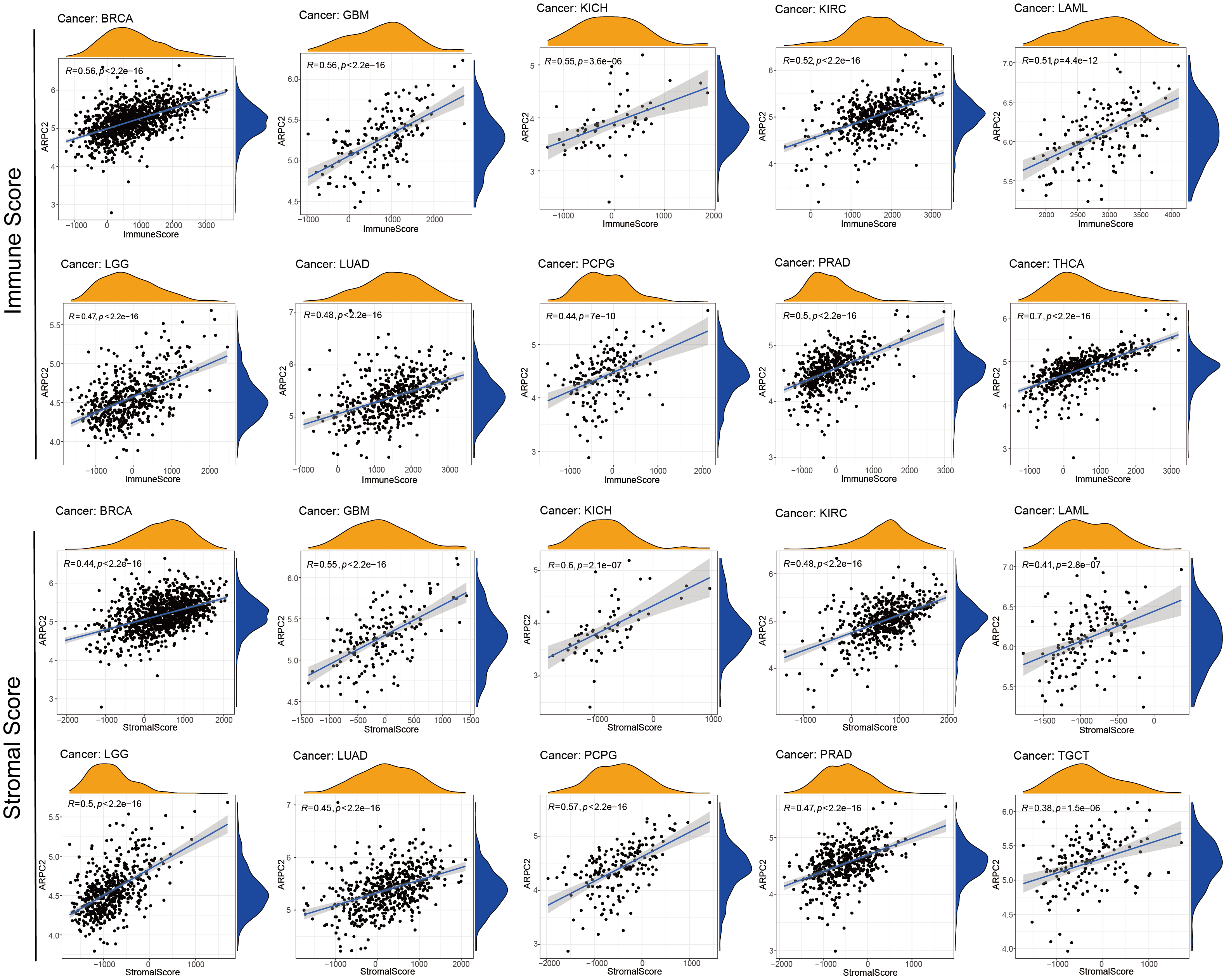

Supplement: Supplementary file 2 [file Image3.TIF]

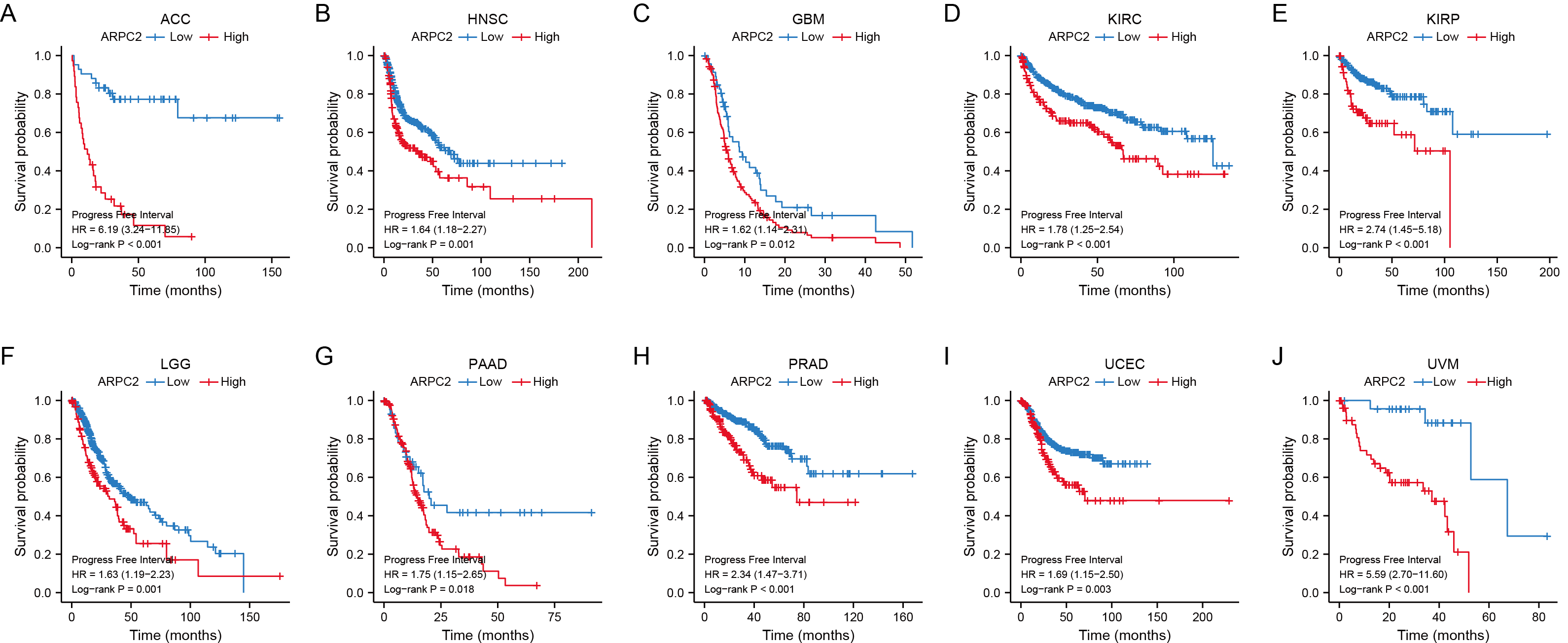

Supplement: Supplementary file 3 [file Image2.TIF]

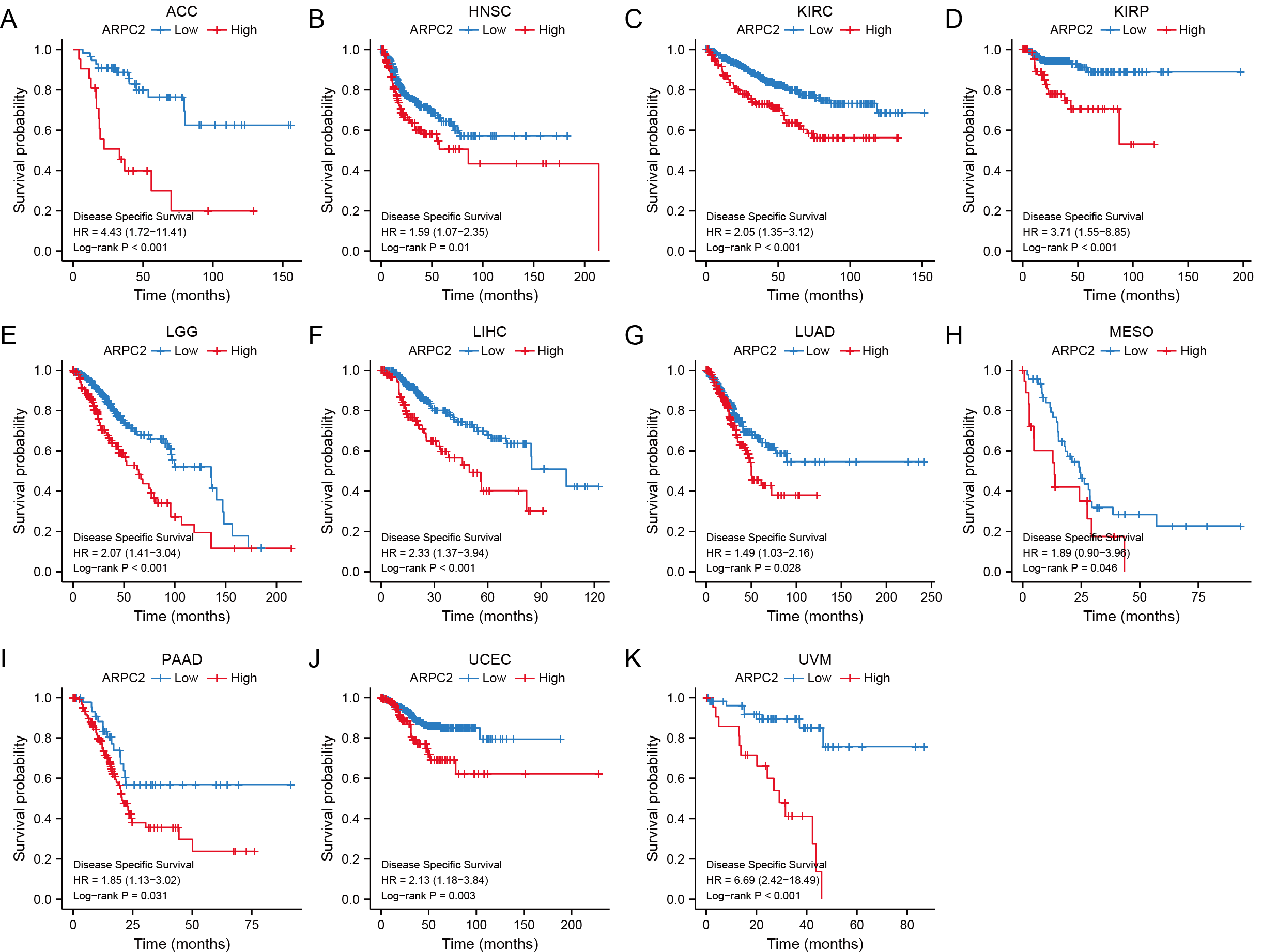

Supplement: Supplementary file 4 [file Image1.TIF]
